# Circulating tumor DNA in early response assessment and monitoring of advanced colorectal cancer treated with a multi-kinase inhibitor

**DOI:** 10.18632/oncotarget.24879

**Published:** 2018-04-03

**Authors:** Caroline Vandeputte, Pashalina Kehagias, Hakim El Housni, Lieveke Ameye, Jean-François Laes, Christine Desmedt, Christos Sotiriou, Amélie Deleporte, Francesco Puleo, Karen Geboes, Thierry Delaunoit, Gauthier Demolin, Marc Peeters, Lionel D’Hondt, Jos Janssens, Javier Carrasco, Raphaël Marechal, Maria Gomez Galdon, Pierre Heimann, Marianne Paesmans, Patrick Flamen, Alain Hendlisz

**Affiliations:** ^1^ Gastro Intestinal Oncology Unit, Medical Oncology, Institut Jules Bordet, Brussels, Belgium; ^2^ Department of Medical Genetics, Hôpital Erasme-ULB, Brussels, Belgium; ^3^ Data Centre, Institut Jules Bordet, Brussels, Belgium; ^4^ OncoDna, Gosselies, Belgium; ^5^ Breast Cancer Translational Research Laboratory, Institut Jules Bordet, Université Libre de Bruxelles, Brussels, Belgium; ^6^ Service of Digestive Oncology, Universitair Ziekenhuis Gent, Gent, Belgium; ^7^ Oncology Department, Hôpital de Jolimont, La Louvière, Belgium; ^8^ Gastroenterology Department, Centre Hospitalier Chrétien St-Joseph, Liège, Belgium; ^9^ Oncology Department, Universitair Ziekenhuis Antwerpen, Antwerpen, Belgium; ^10^ Oncology Department, Centre Hospitalier Universitaire, UCL Namur (site de Godinne), Dinant, Belgium; ^11^ Department of Gastroenterology, AZ Turnhout, Turnhout, Belgium; ^12^ Oncology Department, Grand Hôpital de Charleroi, Charleroi, Belgium; ^13^ Department of Gastroenterology, GI Cancer Unit, ULB-Erasme, Brussels, Belgium; ^14^ Department of Pathology, Jules Bordet Institute, Free University of Brussels, Brussels, Belgium; ^15^ Nucleair Medicine Imaging and Therapy Department, Institut Jules Bordet, Brussels, Belgium

**Keywords:** early-response, biomarker, multi-kinase inhibitor, colorectal cancer, ctDNA

## Abstract

Predictive biomarkers are eagerly awaited in advanced colorectal cancer (aCRC). Targeted sequencing performed on tumor and baseline plasma samples in 20 patients with aCRC treated with regorafenib identified 89 tumor-specific mutations of which ≥50% are also present in baseline plasma. Droplet digital PCR (ddPCR) assays were optimized to monitor circulating tumor DNA (ctDNA) levels in plasmatic samples collected throughout the treatment course and showed the importance of using the absolute value for ctDNA rather than the mutant/wild type ratio in monitoring the therapy outcome. High baseline cell free DNA (cfDNA) levels are associated with shorter overall survival (OS) (HR 7.38, P=0.001). An early increase (D14) in mutated copies/mL is associated with a significantly worse PFS (HR 6.12, P=0.008) and OS (HR 8.02, P=0.004). These data suggest a high prognostic value for early ctDNA level changes and support the use of blood-born genomic markers as a tool for treatment.

## INTRODUCTION

Regorafenib (Stivarga^®^, Bayer AG, Leverkusen, Germany) is one of the therapeutic options for patients with chemorefractory advanced colorectal cancer (aCRC). It is associated with a small overall survival (OS) improvement and significant toxicities including hand foot skin reactions, diarrhea, hypertension and fatigue [1]. Hence, there is a need to develop tools able to quickly identify patients unlikely to benefit from the treatment in order to spare them from unnecessary side effects.

Circulating cell-free DNA or cfDNA is currently under wide investigation as a potential biomarker for tumor response. Identification of cfDNA indicates the presence of extracellular nucleic acids in the circulation due to apoptosis or necrosis. Deriving from normal cell types and primary tumor cells, circulating and metastatic tumor cells, it is present in healthy individuals as well as in cancer patients, although usually in higher quantities in the latter [2]. The amount of cfDNA varies strongly and may overlap between healthy and cancer populations. In addition, metastatic cancer patients usually have higher cfDNA levels than the non-metastatic ones. Several studies have already described the inverse correlation between cfDNA levels and overall survival [3, 4]. In 166 aCRC patients treated with regorafenib, Tabernero *et al.* showed that a high cfDNA baseline concentration was associated with shorter progression-free survival (PFS) and OS compared to patients with a lower cfDNA plasma concentration [5]. A fraction of the cfDNA in cancer patients is circulating tumor DNA (ctDNA), which represents the DNA liberated by the tumor cells after apoptosis or necrosis. Because ctDNA is released into the bloodstream, plasma samples can be used as “liquid biopsies”, allowing the detection and follow-up of tumor-specific anomalies [2, 6, 7].

Recent developments in molecular technologies offer the possibility to screen the tumor molecular landscape in a relatively timely and cost effective manner [8]. The screening can be performed on tumor tissue collected during surgery, from a biopsy or on tumor DNA extracted from a patients’ plasma. Bettegowda and colleagues evaluated the effectiveness of ctDNA detection in a large cohort of 640 cancer patients by digital PCR technologies. Briefly, ctDNA was detected in more than 75% of patients with different solid tumor types including colorectal, ovarian, advanced pancreatic, gastroesophageal, bladder, breast, hepatocellular, melanoma, and head and neck [4], while more than half of patients with primary brain, prostate, renal or thyroid cancer do not exhibit detectable levels of ctDNA with current methods.

The assessment of ctDNA represents an emerging field of cancer research. Although ctDNA cannot be used to identify and locate the tumor origin, it might be an excellent technique to monitor events over time. Repeated measurements of ctDNA will be easily preferred over multiple tumor biopsies, which are invasive, expensive and often difficult to repeat. The proportion of ctDNA in cfDNA varies greatly, between 0.01% to more than 90%, depending mainly but not only on disease staging, pushing the sensitivity boundaries of our current technologies [9, 10]. However, sensitivity of sequencing techniques is continuously improving and one assumes that these techniques will also be applied in situations with low levels of ctDNA (early-stage disease, minimal residual disease…) in the near future. Tie *et al.* recently published the first data on ctDNA fot the detection of residual disease in 230 patients with stage II colon cancer. They were able to show that ctDNA detection after colon resection identifies patients with high risk of disease recurrence [11].

With the exception of *RAS* mutations, up to now, knowledge on tumor genetics hardly influences the management of CRC and the understanding of the tumor history at a personalized level [12].

**Figure 1:**
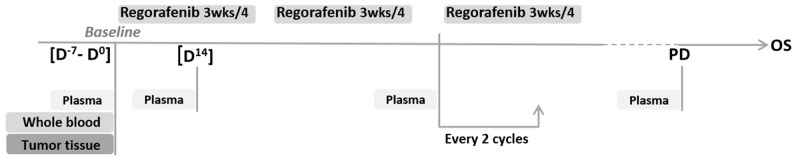
Sample collection in the RegARd-C study

Taking into account the relatively narrow efficacy/toxicity ratio for regorafenib in aCRC, and the lack of available biomarkers, the initial purpose of the clinical trial RegARd-C (NCT01929616) was to detect and analyze tumors unlikely to benefit from regorafenib using combined early metabolic assessment of response and modern biological armamentarium. The sample collection in RegARd-C is depicted in Figure 1.

We present hereunder the results of a small exploratory study on 20 patients included in the RegARd-C trial for which we had all necessary samples available at the time of analysis, aiming (i) to correlate the findings of targeted gene sequencing for known oncogenes and tumor suppressor genes between tumor tissue and ctDNA in plasma, (ii) to cross-examine these findings in plasma samples with the use of the droplet digital PCR (ddPCR) technology and (iii) to assess the use of serial plasma samples to detect quantitative changes in those tumoral genomic biomarkers that would reflect on resistance to the drug [13].

The time-points selected for sample collection and the methods used for sample analysis open a window of opportunities in the search for successful biomarkers. More specifically, these biomarkers could define general therapeutic possibilities and early response assessment at the level of each individual patient, reflecting the very basic principles of personalized medicine. To the best of our knowledge, this study is the first to assess ctDNA as a potential early (after 14 days of treatment) biomarker for response assessment in aCRC with potential direct implications in clinical practice.

## RESULTS

### Targeted gene sequencing analysis

Mutations in 47 important target genes for CRC were identified in 100% of patients with an average of 5 mutations per patient (range 1-10). A total of 89 mutations were identified in the tumor tissue, consisting of 81 substitutions, 3 insertions and 5 deletions. Concordant with literature, the most common mutations were found in *APC* (70% of patients), *TP53* (70%), *KRAS* (70%) and *PIK3CA* (40%) [8]. Patient characteristics and results are described in Supplementary Tables 1 and 2, respectively. Interestingly, in 1 patient we found both a variant in *KRAS* and in *BRAF*, although these variants have frequently been described as being mutually exclusive [14]. We also confirmed the presence of both mutations by conventional PCR. Discordance in *KRAS* status as assessed by targeted gene sequencing or conventional PCR at diagnosis was seen in 15% of the patients. More specifically, 1 patient was previously diagnosed with a KRAS mutation on codon 13 as part of the routine analysis, while this mutation was found neither by targeted gene sequencing nor by our in-house validated conventional PCR, which means that this patient would have had the opportunity to potentially benefit from anti-EGFR therapy after failure of the study medication. On the contrary, a *KRAS* mutation was detected by targeted gene sequencing (and confirmed with our in-house PCR) in two other patients previously diagnosed as being *KRAS*-wild-type. To reduce the variability in mutation detection due to tumor heterogeneity, analysis was always performed on the same FFPE tissue block (Supplementary Table 3).

Subsequently, via sequencing, we investigated whether these tumor-specific mutations were also detectable in plasma at baseline and at day 14, during the first regorafenib treatment cycle. Results for each patient are described in Supplementary Table 2. In summary, in 12 out of 20 patients (60%) all tumor tissue-specific mutations were also present (variant allele frequency, VAF>1%) in plasma at baseline. In 7 out of 20 patients (35%) tumor tissue-specific mutations were partially present in plasma at baseline. In 1 patient (RGR-54) none of the tumor tissue-specific mutations were found in any of the plasma samples (VAF>1%). In 4 out of 20 patients (20%) (RGR-2,28,35,43) tumor-specific mutations present in the baseline plasma sample could not be retrieved by sequencing in plasma after 2 weeks of regorafenib therapy (VAF>1%). 2 out of 20 patients (10%) (RGR-44, 58) had a mutation in plasma at baseline that was not detected in the tumor tissue.

### Droplet digital PCR technology evaluation

**Figure 2:**
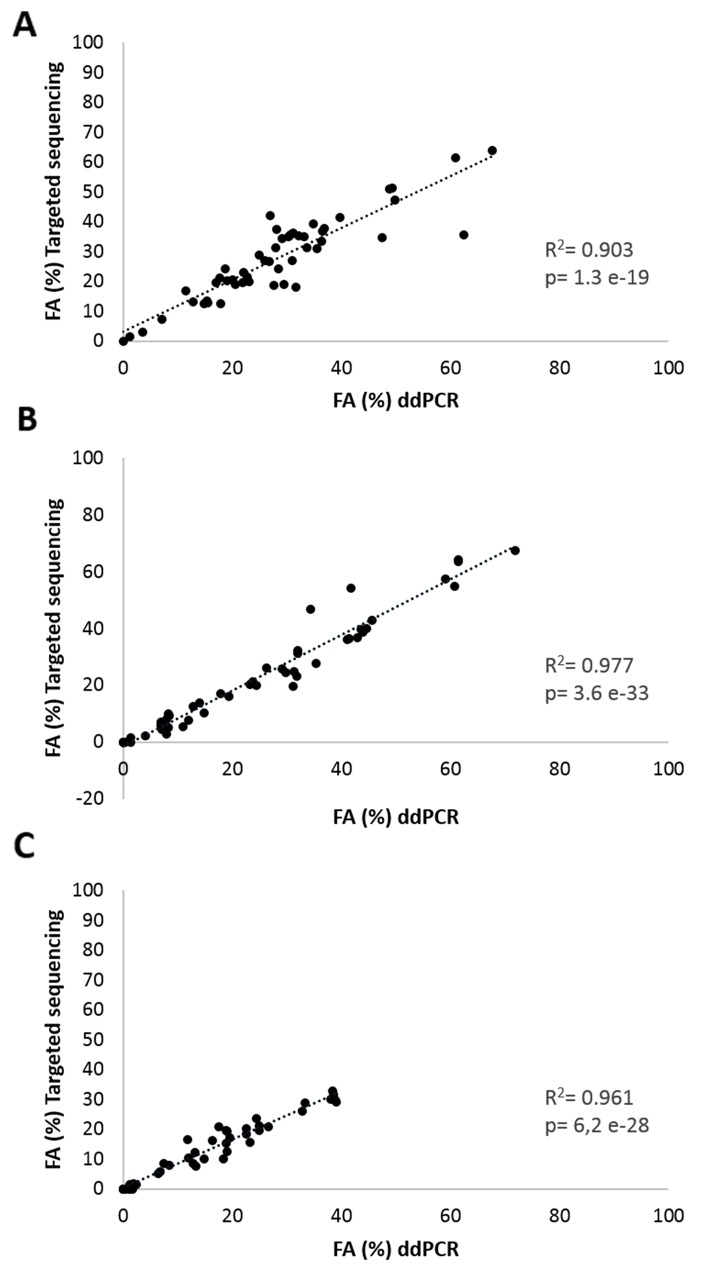
Correlation analyses of mutation frequency Fractional abundance (FA%= mutated copies/ mutated copies + wild type copies) of all targeted mutations obtained by targeted sequencing and ddPCR in **(A)** FFPE tumor samples, **(B)** in plasma samples at baseline and **(C)** in plasma samples at 14 days after start of regorafenib treatment of 19 aCRC patients.

The sequencing results were subsequently validated with the ddPCR technology. On average 3 mutations (range 1-4) were selected per patient based on the highest VAFs. All data are shown in Table 1, except for patient RGR-7 for which ddPCR raw data could not be interpreted due to a lack of generated droplets by ddPCR. A strong correlation between sequencing and ddPCR results in terms of fractional abundance (FA) was observed for tumor samples (R^2^=0.903, P=1.3 e^-19^), for plasma at baseline (R^2^=0.977, P=3.6 e^-33^) and for plasma at C1 (cycle 1 of regorafenib treatment) (R^2^=0.961, P=6.2 e^-28^). The scatterplots are shown in Figure 2.

**Table 1:** Results of targeted gene sequencing analysis compared to ddPCR analysis on archived tumor samples and plasma samples at baseline (BL) and after 14 days (D14) of regorafenib therapy (cycle 1). Patient RGR-7 was excluded due to a poor quality blood sample at BL and D14. VAF, variant allele frequency

Patient	Gene	Mutation	Variant	Targeted sequencing			ddPCR		
				VAF (%) tumor	VAF (%) plasma baseline	VAF (%) plasma D14 (C1)	VAF (%) tumor	VAF (%) plasma baseline	VAF (%) plasma D14 (C1)
RGR-1	APC	p.R213^*^	c.637C>T^2^	16,83	26,13	16,55	11,4	26,2	11,84
	KRAS	p.A146V	c.437C>T^2^	26,84	57,47	20,72	26	59,1	26,6
	TP53	p.R175H	c.524G>A^2^	34,94	67,46	28,94	33,2	71,8	33,4
RGR-2	APC	p.Y1376^*^	c.4128T>A^2^	35,55	2,05	<1	62,4	4	0
	KRAS	p.G12V	c.35G>T^2^	18,97	WT	WT	29,5	1,3	0,09
RGR-4	BRAF	p.V600E	c.1799T>A^2^	19,51	21,12	9,95	21,9	23,8	14,8
	KRAS	p.G12D	c.35G>A^2^	7,14	WT	WT	7,14	0,05	0,04
	PIK3CA	p.H1047R	c.3140A>G^2^	36,24	36,18	23,55	31,1	41,1	24,4
RGR-7	PIK3R1	p.S102^*^	c.305C>A^2^	19,08	44,53	49,27	20,6	44,8	-
	TP53	p.H214R	c.641A>G^3^	24,16	42,55	30,6	18,1	-	-
RGR-14	KRAS	p.G12D	c.35G>A^2^	36,62	24,69	10,05	36,6	31,4	18,3
RGR-24	APC	p.S1032^*^	c.3095C>A^2^	18,73	54,17	12,58	27,6	41,7	19,1
RGR-26	KRAS	p.G12S	c.34G>A^2^	28,77	15,99	8,61	24,9	19,3	12,9
	TP53	p.R196^*^	c.586C>T^2^	37,5	20,32	12,35	28,1	23,3	13,2
RGR-28	SMAD3	p.F343L	c.1029C>A	21,19	5,72	<1	17,7	7	0,98
	TP53	p.R273H	c.818G>A^3^	34,5	5,36	<1	47,5	11	1,7
RGR-30	NRAS	p.G12V	c.35G>T^2^	51,04	42,93	26	48,9	45,6	32,8
	APC	p.W685^*^	c.2054G>A^2^	42,01	32,34	19,63	27	32	18,8
	TP53	p.G245S	c.733G>A	51,35	38,5	15,61	49,4	44	23,3
RGR-35	KRAS	p.G12C	c.34G>T^2^	13,21	7,9	<1	12,8	7,9	1,34
	PIK3CA	p.E545K	c.1633G>A^2^	12,74	9,93	1,49	15,5	8,2	1,74
	TP53	p.R248Q	c.743G>A^3^	19,51	9,27	1,13	17	8,5	1,21
RGR-38	APC	p.E941^*^	c.2821G>T^2^	21,28	5,03	1,54	22,7	8,2	2,4
	KRAS	p.G12D	c.35G>A^2^	19,94	2,78	1,64	23,1	8	1,9
RGR-43	NRAS	p.G12D	c.35G>A^2^	26,49	4,34	<1	26,8	7,1	0,27
	APC	p.R283^*^	c.847C>T^2^	18,03	5,95	<1	31,6	7	0,15
	TP53	p.R248Q	c.743G>A^3^	37,72	7,11	<1	36,9	6,9	0,29
RGR-44	KRAS	p.Q61H	c.183A>C^2^	WT	12,54	10,4	WT	12,8	12
	PIK3CA	p.F83L	c.247TTT>AAA	31,23	36,5	30,04	33,7	41,4	38
	APC	p.R232^*^	c.694C>T^2^	12,57	1,5	1,46	14,87	1,3	1,19
RGR-46	KRAS	p.G13R	c.37G>C^2^	35,04	7,66	5	30,3	11,9	6,36
	PIK3CA	p.E545K	c.1633G>A^2^	39,22	13,91	8,48	34,9	14	7,42
	APC	p.Q1429^*^	c.4285C>T^2^	34,28	10,15	5,72	29,1	14,8	6,8
RGR-50	PIK3CA	p.E542K	c.1624G>A^2^	20,42	31,06	16,06	20,1	32	16,4
	KRAS	p.G12V	c.35G>T^2^	22,79	54,82	29,03	22	60,8	39,1
	TP53	p.R110P	c.329G>C	24,14	39,57	19,4	28,4	43,6	19
RGR-51	KRAS	p.G12A	c.35G>C^2^	35,34	25,84	16,99	32,2	29,1	19,6
	APC	p.Q1429^*^	c.4285C>T^2^	35,43	24,32	15,41	30,7	29,8	18,87
	TP53	p.G245S	c.733G>A	47,14	36,86	21,07	49,8	43	25
RGR-54	KRAS	p.G13D	c.38G>A^2^	12,36	<1	<1	17,8	0,29	0,2
	PIK3CA	p.G1049R	c.3145G>C	13,48	<1	<1	15,4	0	0,013
	APC	p.R232^*^	c.694C>T^2^	20,13	<1	<1	19	0,06	0,1
RGR-56	TP53	p.R337C	c.1009C>T	41,4	39,92	18,22	39,8	44,7	22,6
	APC	p.Q1367^*^	c.4099C>T^2^	27,06	19,68	7,54	31	31,1	13,4
	NOTCH1	p.A1104T	c.3310G>A	31,09	16,87	7,9	27,9	17,8	8,4
RGR-58	KRAS	p.G12C	c.34G>T^2^	2,86	63,42	20,94	3,6	61,4	17,6
	TP53	p.M237K	c.710T>A^2^	1,47	64,11	19,59	1,16	63,4	24,9
RGR-61	APC	p.A1492Cfs^*^1513	c.2510_2511insT^2^	30,85	19,9	20,3	35,6	24,5	22,5
	KRAS	p.Q61H	c.183A>C^2^	33,36	23,27	32,9	36,3	31,8	38,4
	TP53	p.R158H	c.473G>A	63,7	27,62	29,85	67,6	35,3	38,9
	FBXW7	p.A422Qfs^*^443	c.1262delC	61,42	46,72	31,5	60,9	34,3	38,5

**Figure 3:**
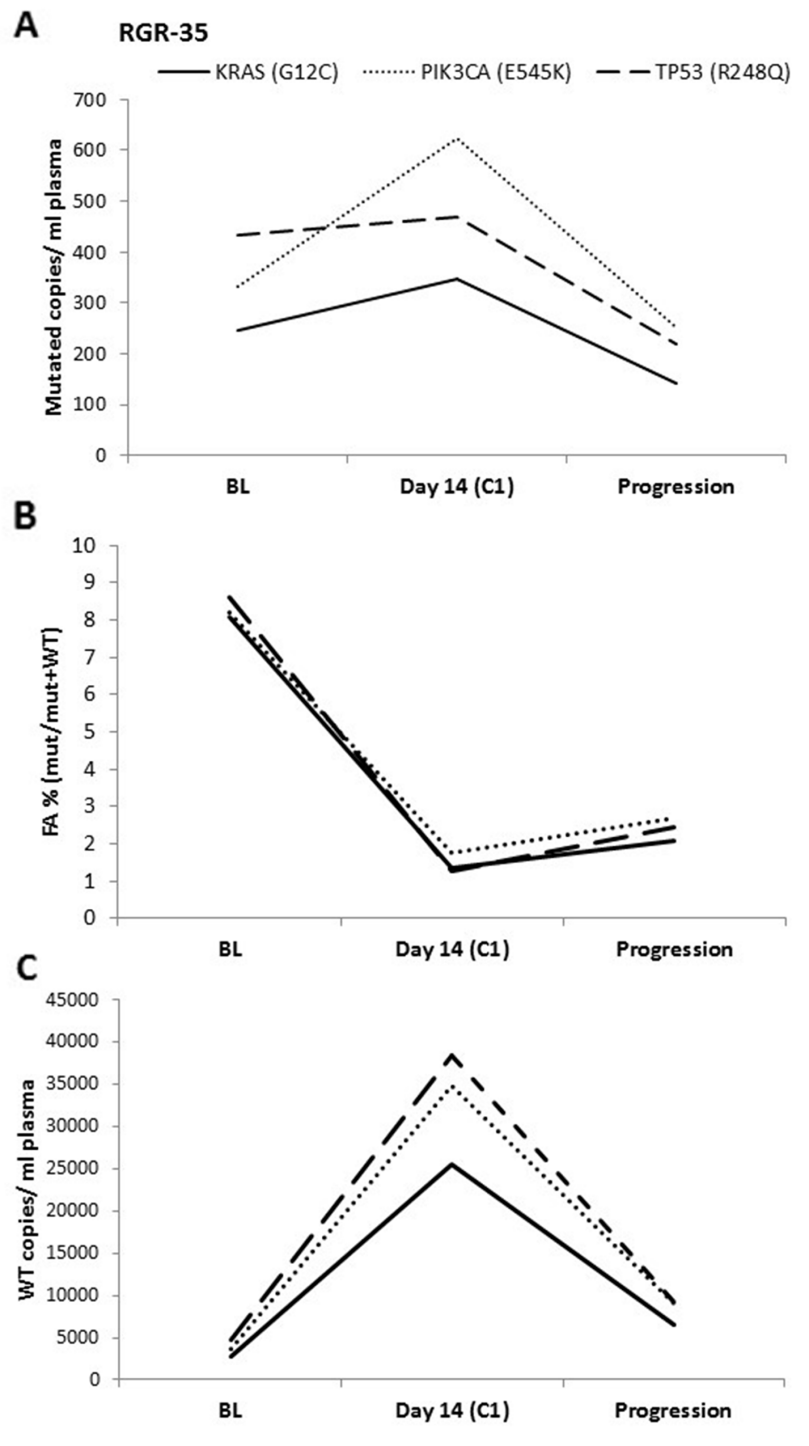
ddPCR data analysis The ddPCR results for one patient expressed as **(A)** mutated copies/ mL of plasma, **(B)** FA (mutated copies/wild-type copies + mutated copies) and as **(C)** Wild-type copies/ mL of plasma. In (A), an increase is seen in mutated copies/ mL between baseline (BL) and at day 14 of cycle 1 (C1). In contrast, a clear decrease is observed when using the FA. This difference can be explained by the 8-fold increase in WT copies between BL and C1.

We then followed patients’ mutations throughout the course of their experimental treatment using ddPCR analysis of plasma samples. Importantly, the interpretation of the results based on ddPCR was heavily influenced by the way the data were represented. More specifically, ddPCR results can be expressed (i) as usually reported in the literature by FA, denoting the proportion of the mutant allele frequencies (mutated copies/wild-type copies + mutated copies while presuming that wild-type copies are constant over time) or (ii) by absolute value (mutated copies/ mL of plasma). In all patients, ddPCR detected a strong increase of the number of wild-type copies in plasma during the first cycle of regorafenib, thereby directly affecting the FA. To avoid the possible impact caused by changes in wild-type copy numbers, we conducted our experiments using the mutated copies/ mL of plasma variable over time. The impact of wild-type copy variations on FA and thus on the patient’s dynamic monitoring is illustrated in Figure 3.

### ctDNA monitoring in patients treated with regorafenib therapy

**Figure 4:**
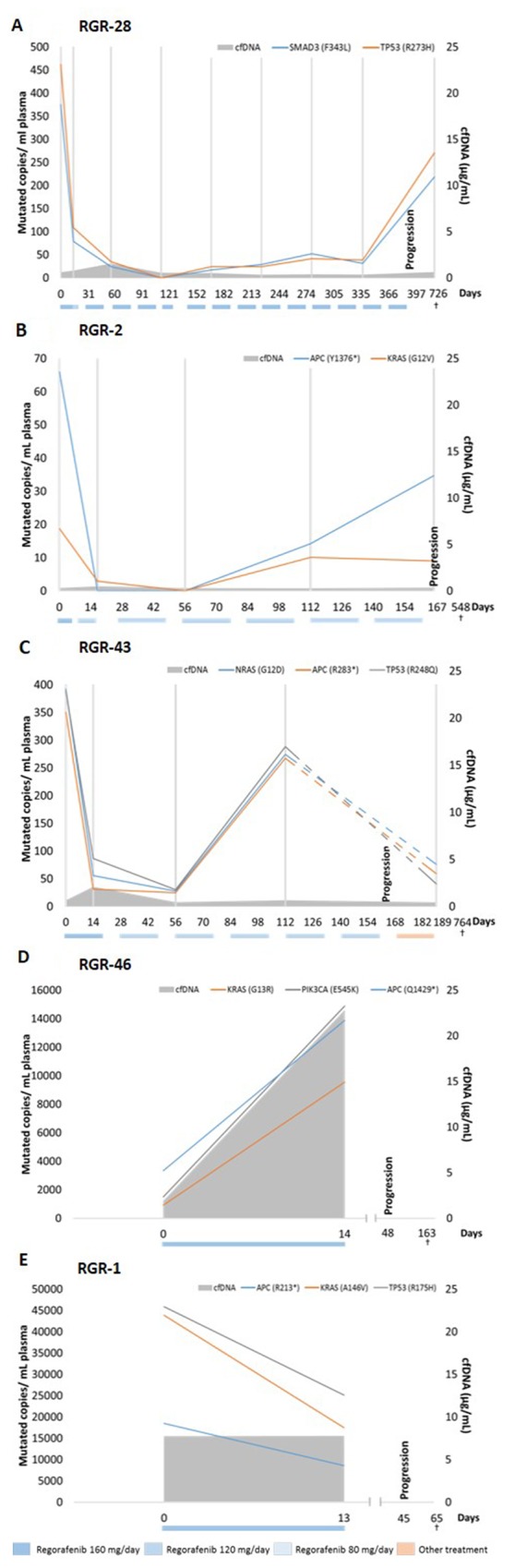
ctDNA and cfDNA status before and during regorafenib treatment ctDNA concentrations and cfDNA for 5 patients that received regorafenib therapy. Colored boxes below the graphs indicate the dose and the period during which treatment was administered. Grey vertical lines indicate the time-points of ctDNA analysis. Patient **(A)** shows an immediate drop (4-fold) in ctDNA levels at day 14. A 6-fold increase is detected at progression. The patient denoted in **(B)** also shows an immediate decrease in ctDNA at day 14 and a significant increase in ctDNA at least 48 days before clinical disease progression. Interestingly, both represented mutations behave differently upon disease progression. **(C)** After an immediate drop in ctDNA, an increase is detected at least 56 days before clinical progression. Interestingly, 6 days after progression this patient received FOLFOX therapy explaining the drop (dashed lines) in ctDNA levels between 2 ctDNA measurements surrounding the time-point of progression. In contrast, the patient in **(D)** showed a 4-fold increase in ctDNA for all clones immediately after starting the regorafenib treatment. The patient progressed clinically 48 days after the start of treatment. In parallel, a 12-fold increase is measured in total cfDNA between baseline and 14 days after the start of treatment. Patient RGR-1 in **(E)** shows a high cfDNA level at baseline of 23.2μg/mL. In contrast, all measured mutations in ctDNA decrease between baseline and day 14 after start of regorafenib therapy. The colored boxes below the graphs indicate the dose and the period during which the treatment was administered.

Next, we analyzed whether plasma DNA could be used to detect patterns of resistance to treatment by monitoring the ctDNA of the 20 studied patients before the start of regorafenib, during the first therapeutic cycle and then every 2 cycles until disease progression. Serial ctDNA concentrations of 5 randomly selected patients are represented in Figure 4. All other patient graphs are shown in Supplementary Figure 1. The time course of patient RGR-28 shows an immediate 4-fold drop in ctDNA levels at the beginning of treatment (Figure 4A). A 6-fold increase is detected just before clinical progression. An immediate decrease is also detected in patient RGR-2 at the beginning of treatment and a significant increase is seen in ctDNA at least 55 days before clinical disease progression (Figure 4B). In this patient the kinetics of mutational levels observed between 2 mutations such as APC (Y1376^*^) and KRAS (G12V) were different upon disease progression, representing most probably that, although all clones have the APC mutation, KRAS-mutated and KRAS-wild-type subclones behave differently during treatment. Patient RGR-43 demonstrated an immediate drop in ctDNA during treatment, followed by an increase at least 57 days before clinical progression for all investigated mutations (Figure 4C). The important decline in ctDNA levels between the 2 ctDNA measurements surrounding the time-point of progression can be explained, by the fact that this patient received combined cytotoxic chemotherapy with oxaliplatin, 5 Fluorouracil and folinic acid (FOLFOX) after disease progression but before plasma collection of circulating DNA. In contrast to the first three patients, patient RGR-46 showed a 4-fold increase in ctDNA for all mutations immediately after starting the regorafenib treatment. This patient progressed clinically 48 days after the start of the treatment (Figure 4D). Serial carcinoembryonic (CEA) levels were measured as part of standard clinical practice in half of the patients, but could not be correlated with the ctDNA evolution, as the time-points of blood sample collection and analysis differed.

### Circulating DNA dynamics and prognosis

**Figure 5:**
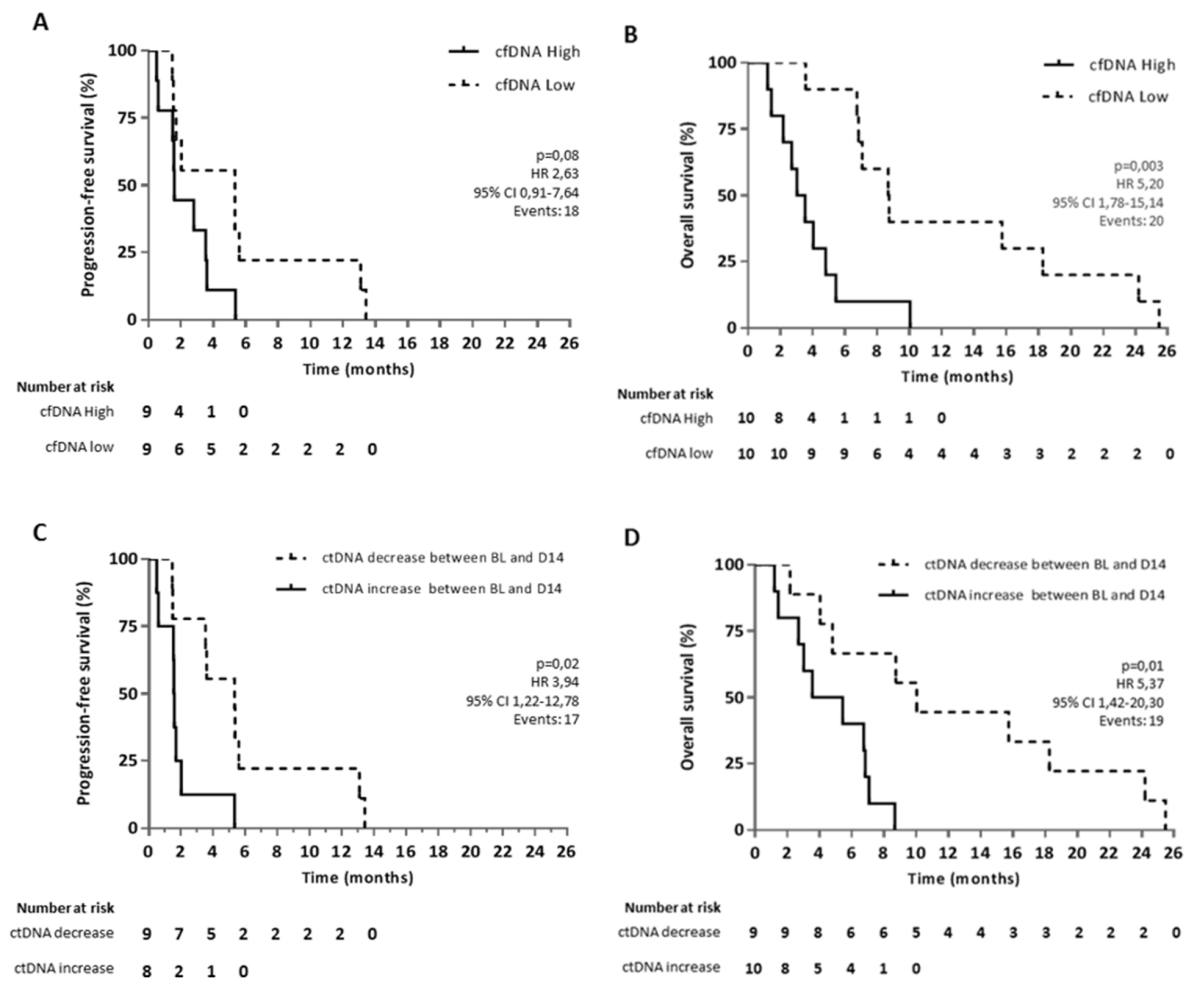
Kaplan-Meier curves for OS and PFS in aCRC patients receiving regorafenib therapy Survival curves are represented according to the level of **(A, B)** cfDNA in plasma at baseline for OS and PFS, respectively and according to **(C, D)** the increase or decrease of ctDNA levels between baseline (BL) and 14 days after the start of regorafenib therapy (D14). Two patients out of 20 were not included in the PFS analysis due to missing information on clinical progression. For ctDNA analysis, another patient was excluded due to a poor quality blood sample at C1.

Based on previous literature, we investigated whether baseline cfDNA levels were inversely correlated to PFS and OS. We measured the total amount of circulating free DNA, including the circulating non-tumoral DNA as well as the circulating tumor DNA cfDNA levels. Although there was a trend, no significant difference was seen between high and low cfDNA concentration and PFS (HR 2.63, P=0.08, 95% CI 0.91-7.64) (Figure 5A). However, our findings confirmed that a high baseline cfDNA concentration (higher than median) was associated with a significantly inferior OS compared to patients with a lower baseline cfDNA concentration (lower than median) (HR 5.20, P=0.003, 95% CI 1.78-15.14) (Figure 5B). Also, the continuous univariate analysis per one-unit increase correlated significantly with OS (HR 1.66, P=0.001, 95% CI 1.22-2.24).

Furthermore, we examined if ctDNA dynamics during regorafenib therapy had a prognostic value. We therefore assessed increase (threshold of 0) or decrease in investigated mutated copies by measuring between baseline and day 14 of C1 of regorafenib therapy and correlated these results with PFS and OS. Our analysis showed that an increase of mutated copies per mL of plasma between baseline and day 14 is associated with a significantly worse clinical outcome with a median PFS of 1.6 months versus 5.3 months (HR 3.94, P=0.02, 95% CI 1.22-12.78) and a median OS of 4.5 months versus 10 months (HR 5.37, P=0.01, 95% CI 1.42-20.30) (Figure 5C, 5D). Moreover, we determined by multivariate analysis that a high cfDNA and an increase in ctDNA are independent factors both for PFS (high cfDNA HR 4.19, P=0.02, 95% CI 1.25-14.06, ctDNA increase HR 6.12, P=0.008, 95% CI 1.62-23.09) and OS (high cfDNA HR 7.38, P=0.001, 95% CI 2.19-24.85, ctDNA increase HR 8.02, P=0.004, 95% CI 1.93-33.36). There is no evidence for an association between cfDNA (high/low) and ctDNA (increase/decrease) with P-value=1 or between ctDNA and cfDNA as a continuous variable (P=0.81). An example of this independence of both markers is represented for patient RGR-1 in Figure 4E. This patient shows a high baseline cfDNA level which is in line with his short OS. However, all measured mutations in ctDNA decreased between baseline and 14 days after the start of treatment. Patient RGR-51 shows a high baseline cfDNA level. In contrast, all measured mutations in ctDNA increased between baseline and 14 days after the start of treatment (Supplementary Figure 1J). Since both patient RGR-43 and patient RGR-61 received post-study treatment (respectively, FOLFOX and Panitumumab) which could affect the OS, both patients were removed in a second statistical analysis. Yet, the removal of these patients did not affect the prognostic value of ctDNA nor of cfDNA on OS both in the univariate and multivariate analysis.

## DISCUSSION

Until recently, the measurement of tumoral molecular biomarkers in the context of clinical and therapeutic monitoring has been poorly investigated mainly due to the need of invasive biopsies to obtain serial tumor samples. Also, the detection of tumoral biomarkers was essentially done in the context of resistance to targeted therapies. More importantly, tumor biopsies are prone to sampling error and may not represent the entire tumoral genetic landscape due to tumor genetic heterogeneity and clonal evolution [15]. Therefore, in the first part of the study, we tried to retrieve the mutations detected in the archived tumoral tissue by targeted sequencing in ctDNA plasma samples. If the same mutations were found, ctDNA in plasma samples can be judged as a fair representation of tumor characteristics. The frequency of detected mutations was similar to the one reported in the literature [8]. However, we found a worrisome discordance in 15% of our studied patients for the KRAS status between targeted sequencing and conventional PCR technologies. Those divergences could be linked to the tumoral genetic heterogeneity [16], although the same tissue blocks were used for both analyzes [15]. Also, since different methods with different limits of detection (LOD) were used in KRAS clinical routine mutational analyzes, we assume that lower tumor mutational frequencies than the LOD could have been not detected. Concerning RGR-46, this patient appeared mutated at 35% in our targeted sequencing results while in the external center no KRAS mutation was detected. This discrepancy is presumably due to a non-detectable technical issue during analysis or to an historical patient identification error. KRAS mutation detection methods should be performed using the same standardized guidelines in order to obtain reproducible and reliable results.

Although, in our study the mutations detected in the tumor were fairly represented in the plasma, 5/20 (25%) patients had at least one tumor-specific mutation totally absent in both plasma samples. Treatments given between the time of collection of the archived tumor and the plasma sample at the start of regorafenib may have had an effect on tumor clonal composition and evolution [17] or tumor clones may simply have been released at undetectable levels in the circulation [4]. Also, two new mutations in two patients were found in plasma samples that were not detectable in the archival tumor tissue. These findings as well could be explained by the fact that archived tumor tissue and baseline plasma samples were not collected simultaneously. This could also be linked to the spatial heterogeneity or clonal evolution. *KRAS* mutations could also have pre-existed in few tumor cells, and have expended due to *KRAS*-wild-type tumor resistance to cetuximab and panitumumab therapy. Data are still missing in literature regarding the clinical meaning of appearance/ disappearance of mutations after CRC treatment.

In addition, the sensitivity of the technology used might play a role as illustrated in patient RGR-4 i.e. where a *KRAS* (G12D) mutation was detected by ddPCR in the plasma but, at a very low level, probably below the sensitivity offered by the targeted sequencing technology. The LOD of the used targeted sequencing technology for a given mutant allele is about 1%, which is 10 times higher that the one offered by the ddPCR [18, 19]. In general, the FA measured by ddPCR strongly correlated with targeted sequencing data. The somewhat lower correlation (R^2^=0.90) observed for tumor samples compared to the plasma samples might be explained by the negative impact of fragmented FFPE DNA on ddPCR analysis.

Another important issue raised during our study was the choice of data representation for ddPCR. cfDNA includes both mutant and wild-type DNA derived from the tumor and from non-tumor tissue. Often, ddPCR data as well as targeted sequencing data are represented as FA or as the ratio of mutated alleles over wild-type alleles plus mutated alleles, using the wild-type copies as an intra-patient control for exclusion of poor sample quality or poor sample handling during cfDNA extraction. This data representation would be correct and efficient as long as the wild-type copies are constant over time. Due to the choice of sample collection and cfDNA analysis at 14 days of treatment in our study, it became clear that the treatment may significantly affect the number of wild-type copies through toxicity on normal tissues, introducing a possible bias in the FA variable potentially affecting hugely the molecular monitoring of the patients disease. It is generally believed that cfDNA is released into the circulation by apoptosis and necrosis. Regorafenib is known to be toxic for various tissues including the gastrointestinal tract, skin, and liver. [20] It is reasonable to assume that damaged tissues could be the source of a massive release of wild-type cfDNA in the blood circulation. This deserves further investigation. Since exercise, trauma, pregnancy etc. also affect the release of DNA in the bloodstream, caution should be taken when considering non-mutated cfDNA as a standard for data representation [21, 22]. Pallisgaard and colleagues recently suggested to use an external standardization method by spiking the plasma samples with quantifiable non-human DNA fragments to control for loss of DNA during sample preparation [23]. This proposal should be considered when measuring mutated copies over time.

Our results provide additional valuable information on tumor burden changes during treatment and allow us to correlate the molecular profile of patients with their clinical follow-up. The need to follow several mutant alleles in order to provide a reliable molecular monitoring for each individual patient makes such a strategy difficult to translate into clinical practice as the design and optimization of such personalized assays is time consuming and costly. Also, many but not all detected mutations were followed over time. The average time between an increase in ctDNA in at least one mutant clone and clinical progression should be calculated on a larger patient cohort. In future analyses, a targeted sequencing analysis will be performed at progression in order to try to explore the existence of potentially newly acquired tumoral clones under treatment, or the appearance of tumoral subclones present but not detectable at diagnosis.

Interestingly, in a substantial proportion of patients, an increase in ctDNA burden preceded the clinical or radiological progression. We also confirmed the known inverse correlation between cfDNA at baseline and OS. In contrast to previous literature, the cfDNA measured (in μg/mL) at baseline in this study contains the total amount of circulating free DNA, including the circulating non-tumoral DNA as well as the circulating tumor DNA. Although non-tumor cfDNA can be influenced by variety of reasons [22], the efficacy and speed of our analysis technology should outweigh this limitation and could potentially be more easily translated to the clinical practice, as reinforced by the fact that our results are in line with current literature (measured as genome equivalents of DNA per mL [5]). If this correlation is confirmed in larger cohorts, it would allow for stratification of patients according to a strongly validated prognostic variable, easily accessible for clinicians. This may have a tremendous impact on treatment choices and stratification of patients in future clinical studies. Finally, our analysis showed that an increase of mutated copies per mL of plasma as early as 14 days after the start of treatment is associated with a significantly worse clinical outcome in aCRC patients treated with regorafenib independently from cfDNA levels. To the best of our knowledge, we are the first to demonstrate that the measurement of the mutant allele burden performed before treatment and at 14 days in the context of regorafenib treatment may have a predictive/ prognostic value in aCRC patients paving the way towards an early treatment personalization. Although our preliminary data strongly suggest that the change in ctDNA mutation levels could be used as a tool to predict early-response, this should be confirmed in the whole patients’ population of the RegARd-C study and thereafter in a prospective randomized study. If confirmed, its clinical relevance could also be determined in aCRC patients receiving other treatments than regorafenib.

While our analysis included only 20 patients, they all received the same treatment and were followed in a standardized manner with regular sample collections. Moreover, this preliminary analysis provided highly significant results regardless of the sample size and remaining independent risk factors in multivariate analysis. We are currently validating these very promising data in whole population of patients included in the clinical trial RegARd-C. As serial PET scans were performed at baseline and 14 days after the start of regorafenib, future analyzes will also include the correlation of PET data and molecular analyzes in plasma.

## MATERIALS AND METHODS

### Study design and participants

RegARd-C was a prospective multicentric academic trial with a non-randomized design in patients with aCRC refractory to standard therapy. The study aimed at identifying those patients who draw no benefit from treatment with regorafenib using FDG-PET metabolic imaging. In total 141 patients were included in the study and patients received oral regorafenib once a day for the first 3 weeks of a 4-week cycle starting at a dose of 160 mg/day. The starting dose could be reduced following intolerable side-effects. Patients were monitored until disease progression, unacceptable toxicities or any other reason (study withdrawal, loss for follow-up, death…). Genomic sample analysis was performed on a subgroup of 20 patients (composition of RegARd-C patient subgroup, Supplementary Table 1), selected due to their sample availability. Only patients who signed the study informed consent were considered in this study. The baseline patient demographics and disease characteristics are described in Supplementary Table 1. PFS was calculated as the time till progression/death, whatever occurred first. For cfDNA PFS analysis, 2 patients (RGR-51, 56) were excluded due to missing information on clinical progression. Patient RGR-7 was excluded for ctDNA PFS and OS analysis due to poor quality of the blood samples.

### Sample collection and preparation

Blood samples for plasma extraction samples were collected at baseline and after two weeks of treatment. After that they were obtained every two cycles until disease progression or up to 1 year after the start of therapy. Whole blood was also collected at baseline. Previously obtained formalin fixed paraffin embedded (FFPE) primary (18/20) or metastatic tumor tissue (2/20) was gathered at study entry. Prior to tumor DNA extraction, hematoxylin and eosin stained slides were created from (FFPE) tumoral tissue. These slides were assessed by a pathologist for locating the regions where tumor cellularity was maximal (>50%). These regions were then used for DNA extraction using the QIAamp DNA FFPE Tissue kit (Qiagen, Hilden, Germany). Blood samples, collected in EDTA tubes were centrifuged at 2000xg for 15 minutes at 4°C within 1h of sample collection to separate the plasma from the peripheral blood cells. Next, plasma samples were stored at -80°C until cell-free circulating DNA (cfDNA) extraction was performed of 3mL of plasma using the QIAmp circulating nucleic acid kit (Qiagen). DNA from whole blood was extracted with the QIAamp DNA blood Maxi kit (Qiagen). DNA was quantified using the Qubit^®^ 2.0 fluorometer and the Qubit^®^ dsDNA HS assay kit (Life-Technologies, Gent-Brussels, Belgium). Multiple (3-6) cfDNA extractions and quantifications of plasma were performed for 4 patients to measure the intra-patient extraction variability. No significant differences in cfDNA quantification were seen for either patient.

### Targeted gene sequencing

Mutation analysis of tumor DNA was performed with the Ion Torrent technology (Life Technologies, Carlsbad, CA) available at OncoDNA, Gosselies, Belgium. The tumor DNA and cfDNA from whole blood samples were sequenced with the Onco-CRC panel including a screening of 47 genes classically mutated in CRC (Supplementary Table 4, panel A) with a sequencing coverage of 500x–1000x (Supplementary Table 2). Tumor-specific mutations were defined by comparing mutations founded in tumor and matched whole blood samples, and subsequently tracked in 10 ng of plasma DNA samples (sequencing coverage of 25.000x). Additionally, about 2800 other variants from the 50 most commonly reported oncogenes and tumor suppressor genes were tracked in the plasma (OncoDNA, Gosselies, Belgium) (Supplementary Table 4, panel B). Targeted sequencing detected *KRAS* and *BRAF* mutations were also analyzed with the COBAS^®^ KRAS and COBAS^®^ BRAF mutation tests (Roche Diagnostics Limited, Rotkreuz, Switzerland).

### Droplet digital PCR and data analysis

ddPCR materials and methods as well as the supplementary data were written following the digital MIQE guidelines [24]. DdPCR experiments were performed on the Bio-Rad QX200 ddPCR system (Bio-Rad, Hercules, USA). Briefly, a combination of ddPCR Supermix (no UTP) for probes and specific paired PrimePCR™ ddPCR™ Mutation Detection Assays (Bio-Rad, Hercules, USA) were used to detect and quantify the variants (Supplementary Table 5). Some PrimePCR™ ddPCR™ Mutation Detection Assays were custom designed by Bio-Rad (sequences proprietary to the Bio-Rad company). Prior to the mutation analysis, an optimization process of ddPCR Mutation Detection Assays was done including several steps in order to use the assays with a maximum of reliability and reproducibility between each experiment (Supplementary Materials).

For each assay reaction we maximized the amount of unamplified DNA by using 7.6-223.5 ng/reaction for cfDNA samples and 77.4-454 ng/reaction for tumoral DNA. In each reaction we added 12 μL of ddPCR™ Supermix for probes (no dUTP) (Bio-Rad # 186-3024USA), 1.2 μL of mutation and reference assays and water to reach a final volume of 24 μL. For droplet generation, 20 μL of this ddPCR mixture volume was loaded into each DG8 cartridge well (Bio-Rad #186-4008, USA) along with 70μL of droplet generation oil for probes (Bio-Rad # 186-3030, USA). Next, the cartridge was placed in the QX200™ Droplet generator™ (Bio-Rad) in order to generate approximately 20,000 water-oil emulsion droplets. 40 μL of the emulsion was then carefully pipetted into a 96-well plate, sealed with a PX1™ PCR plate sealer (Bio-Rad) and finally placed in a T100™ thermal cycler (Bio-Rad) using the recommended thermal cycling protocol (Supplementary Table 6). DdPCR data was processed using QuantaSoft V1.7.4 software (Bio-Rad) to obtain the concentration, reported in copies of target/μl of reaction, and fractional abundance (FA) of the mutated alleles in the wild-type background. The resulting copies per μL of reaction were adjusted depending on the volume of ctDNA used (raw data are shown in Supplementary Tables 7 and 8). The threshold that determines if droplets are considered as positive is manually adjusted based on experimental setups wherein the false-positive rate (FPR) was estimated for each PrimePCR™ ddPCR™ Mutation Detection Assay. The FA per reaction is calculated as a percentage (%) of the (number of total mutated copies)/ (number of total mutated copies + number of total wild-type copies). If a sample had too low positive events or a total amount of droplets lower than 10,000, the reaction was repeated at least twice in independent runs to validate the results. For FFPE samples we accepted an amount of droplets starting from 9000. Primary tumor DNA was used as positive control and DNA from whole blood as negative control for each assay reaction in every ddPCR run. Also, a no-template control (NTC) was always included as a negative control for the control assay.

### Statistical analysis

Statistical analyses were performed by using Graphpad Prism 6 and SAS 9.4. Gaussian distributions were determined via the D’Agostino & Pearson omnibus normality test. Depending on the normal distributions, the correlation of targeted gene sequencing data and ddPCR was analyzed using the Pearson correlation coefficient or nonparametric Spearman correlation test. Also the cfDNA was correlated to OS and PFS using the same statistical tests. The univariate and multivariate analysis tests for OS and PFS were performed with the Cox’s proportional hazards model. Kaplan-Meier survival estimates were calculated for each group (decrease or increase in mutational burden between baseline and day 14 of cycle 1 and high or low cfDNA levels). The association between cfDNA (higher/lower than median) and ctDNA (increase/decrease) was measured with the Fisher Exact test and with the Mann-Whitney test for the continuous analysis.

## SUPPLEMENTARY MATERIALS FIGURES AND TABLES










