# Supplementary material for: Circulating tumor DNA in early response assessment and monitoring of advanced colorectal cancer treated with a multi-kinase inhibitor
Source: Oncotarget. 2018 Apr 3;9(25):17756–69. doi: 10.18632/oncotarget.24879 (PMC5915153; doi:10.18632/oncotarget.24879)
Supplement: Supplementary file 1 [file oncotarget-09-17756-s001.pdf]

Circulating tumor DNA in early response assessment and monitoring of advanced colorectal cancer treated with a multi-kinase inhibitor

SUPPLEMENTARY METHODS

Prime PCR ™ ddPCR ™ Mutation Detection Assays optimization and data evaluation

The first step in this process includes the determination of the optimal annealing temperature for each paired Prime PCR™ ddPCR™ Mutation Detection Assay (FAM/HEX). A positive (patient tumor) and negative (patient whole blood) control were run across a thermal gradient (50-60°C) and optimum annealing temperature range were determined based on the separation between four clusters (Supplementary Figure 2). Optimal annealing temperature for each assay is shown in Supplementary Table 5. Secondly, we estimated the False-Positive Rate (FRP) by using 10 wild type DNA samples (no tumoral whole blood) which allowed us to monitor false positives and fix a false positive event threshold for each assay. Next, if the false positive event threshold was high (higher than 3 positive events) we added an optimization step to determine the assay limit of detection (LOD). In this experiment, we ran 2 wild type DNA samples (no tumoral whole blood) and 5 serial dilutions (10, 20, 50, 70, 100 ng/well) of mutation-positive controls (tumor sample) for thresholding and determining the LOD. According to "Rare Mutation Detection, Best practices Guidelines" for Droplet Digital™ PCR by Bio-Rad, we estimated that the LOD is the dilution that shows a statistically significant difference from the negative controls. A No Template Content (NTC) was run in each optimization experiment, as described above to monitor contamination. Among all the assays, 5 of the designed Prime PCR™ ddPCR™ Mutation Detection Assays failed the optimization tests by showing no amplification of the targeted gene (in grey in Supplementary Table 5). Hence, these mutations could not be monitored.

Once Prime PCR™ ddPCR™ Mutation Detection Assays (FAM/HEX) were optimized we tested unknown cfDNA plasma samples. We included in this experiment 1 NTC, 1 mutation-positive control (tumor), 1 wild type control (whole blood) and unknown cfDNA plasma samples to test. Finally, we evaluated our droplet data using QuantaSoft V1.7.4 software (Bio-Rad). Each well was checked for accepted droplets, concentration calls and contamination based on the NTC well. According to optimization data, we manually fixed the FRP threshold depending on the ddPCR™ Mutation Detection Assay used. Then we analyzed data following "Rare Mutation Detection, Best practices Guidelines" instructions for Droplet Digital™ PCR by Bio-Rad. A positive and negative result for a specific Prime PCR™ ddPCR™ Mutation Detection Assay (FAM/HEX) are shown in Supplementary Figure 3.

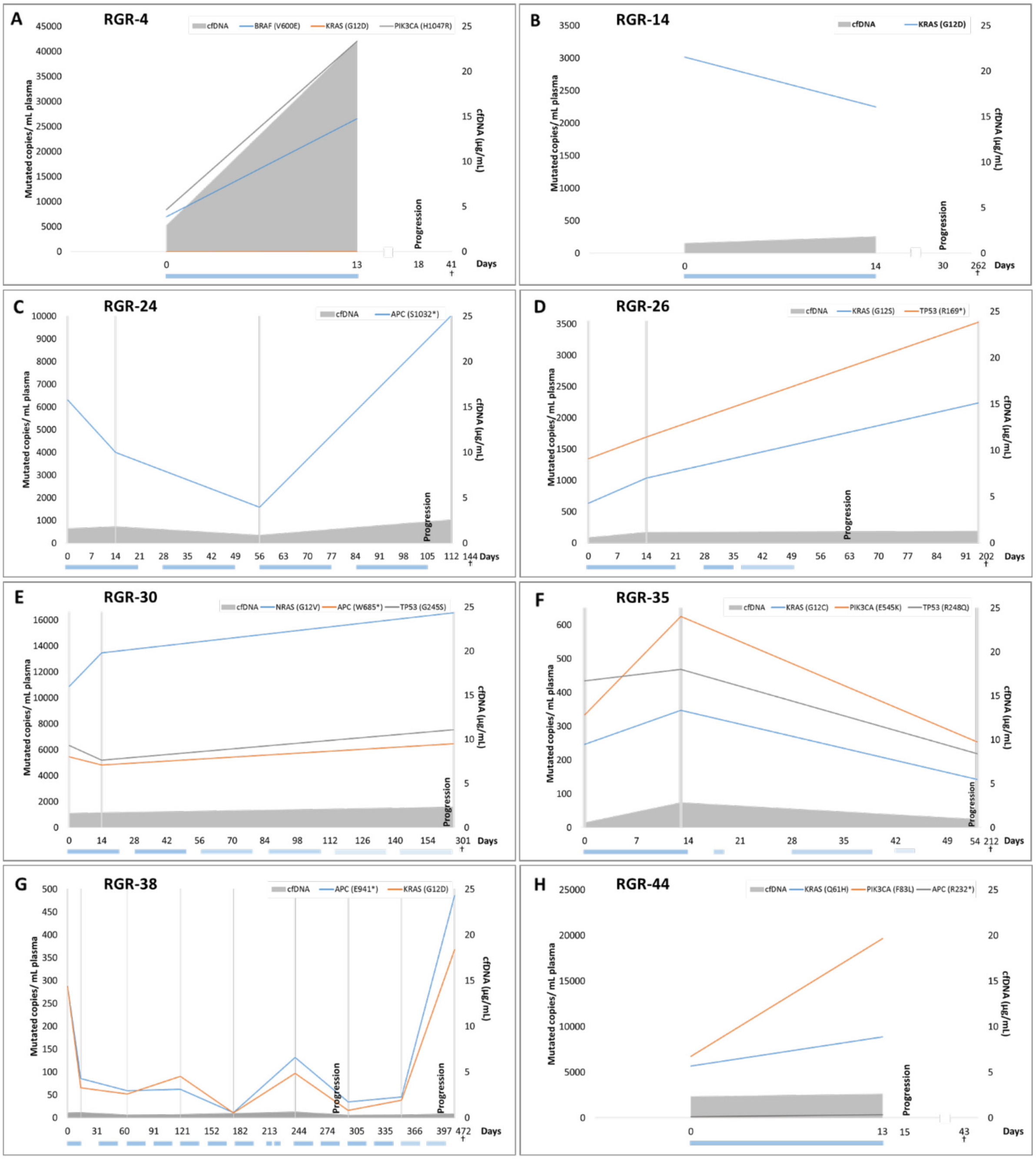

Supplementary Figure 1: ctDNA and total cfDNA status before and during regorafenib treatment. ctDNA concentrations and total cfDNA for 14 patients that received regorafenib therapy. The colored boxes below the graphs indicate the dose and the period during which the treatment was administered. The grey vertical lines indicate the time-points of ctDNA analysis. The grey area represents the cfDNA. The patient **(A)** shows an immediate increase in ctDNA levels for 2 monitored mutations at the start of regorafenib therapy. This patient simultaneously showed an 8-fold increase in cfDNA levels. The patient denoted in **(B)** shows a decrease in ctDNA at day 14. As only 1 mutation was detected via targeted sequencing, this *KRAS* mutation was used for monitoring. After an immediate drop in ctDNA levels in the patient depicted in **(C)**, an increase is detected starting from day 56. The patient in **(D)** shows an increase in ctDNA for all monitored mutations throughout the whole treatment. In **(E)** a different behavior is seen between the different mutations that are monitored as ctDNA of 1 mutation increases immediately during treatment and 2 other mutations decrease initially, but increase also later on. Unfortunately samples have not been collected between day 14 and progression. Patient **(F)** shows a short increase in ctDNA levels and after day 14 an immediate drop. The patient depicted in **(G)** shows an immediate 3-fold drop in ctDNA levels, and ctDNA levels stay relatively low during the course of the disease as well as the cfDNA levels Two clinical progressions were seen in this patient although patient continued with regorafenib treatment after the first progression. A 4-fold increase is measured at the second progression. The patient in **(H)** shows an increase in ctDNA for the majority of the monitored mutations at the start of treatment.

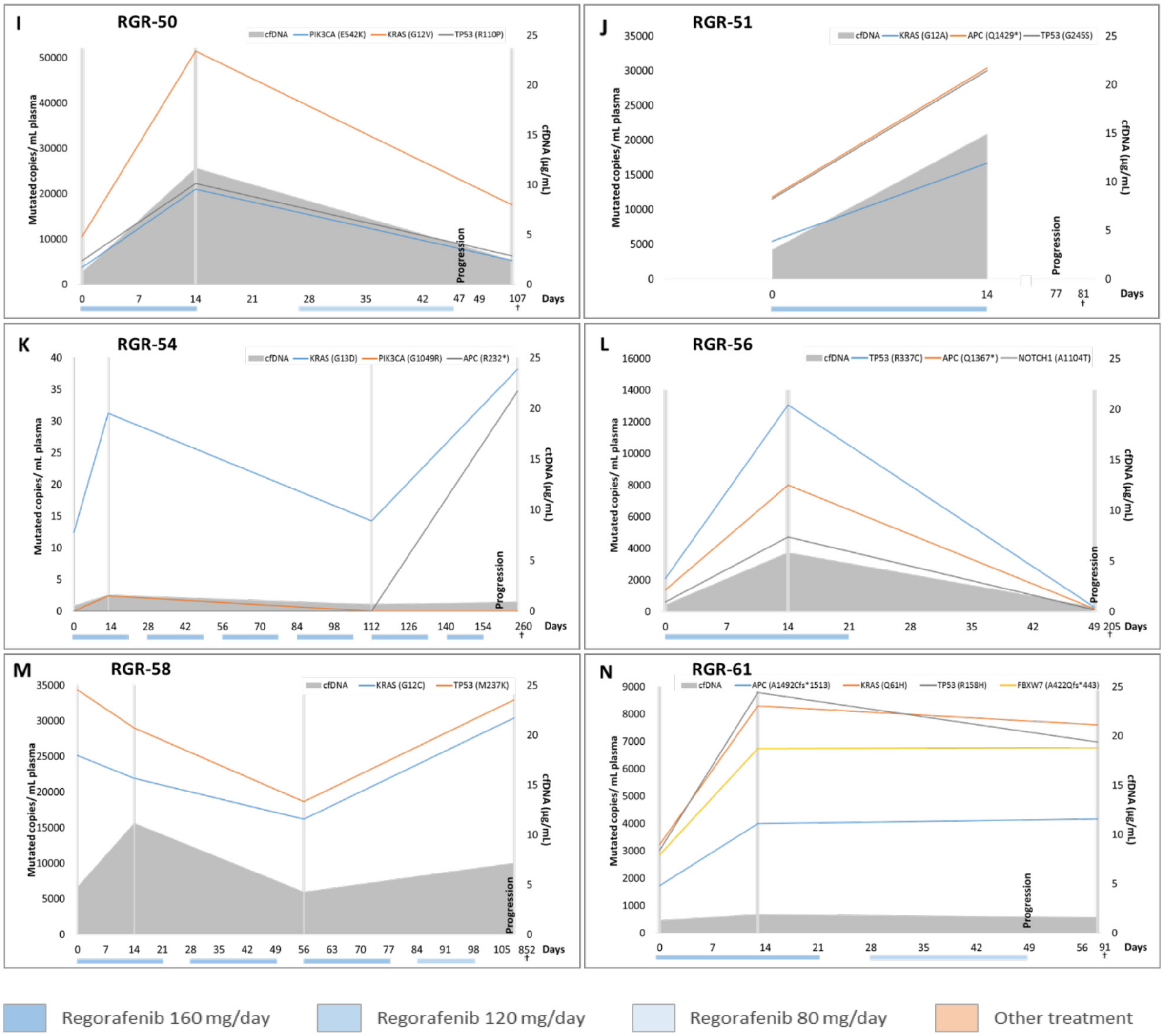

Supplementary Figure 1: ctDNA and total cfDNA status before and during regorafenib treatment. (***Continued***) Patients **(I and L)** show an immediate increase in ctDNA levels, and a decrease after day 14. cfDNA levels follow the same course. The patient **(J)** shows an immediate increase in ctDNA levels for 3 monitored mutations at the start of regorafenib therapy. This patient simultaneously showed a 5-fold increase in cfDNA levels. In panel **(K)** 3 mutations in ctDNA are followed over time. Interestingly, the APC mutation could not be detected at baseline and at day 14, but reappeared later on during treatment and increased expressively. In patient **(M)** cfDNA levels are relatively high throughout the whole course of regorafenib treatment. ctDNA levels decrease initially, but increase after starting the third cycle of therapy. Patient **(N)** show an immediate increase in ctDNA levels, and a relative stabilization between day 14 and disease progression for 4 monitored mutations measured in ctDNA.

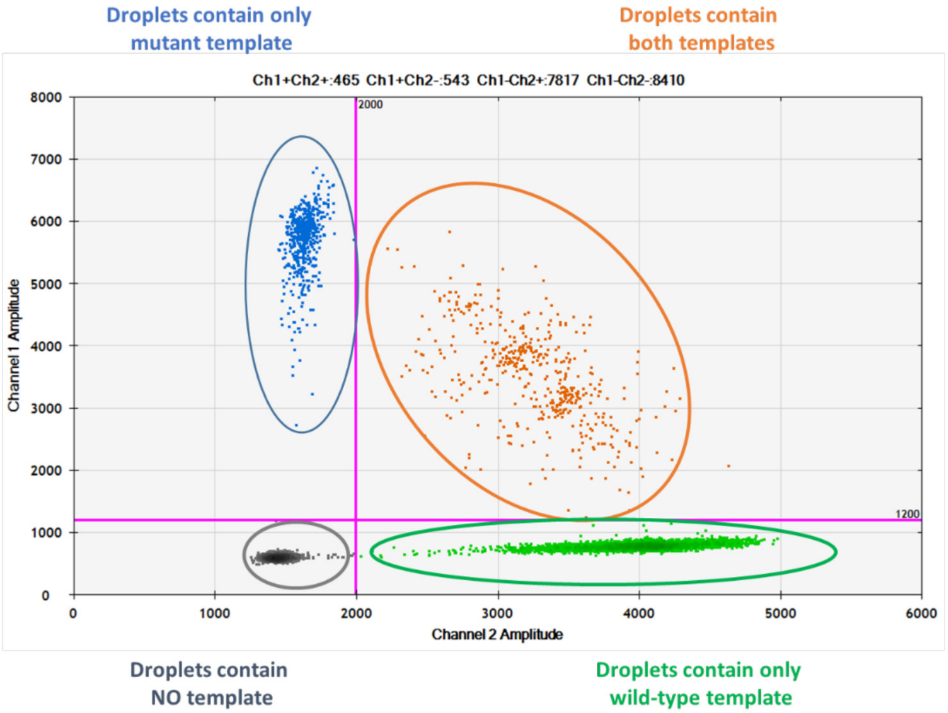

Supplementary Figure 2: Two-dimensional scatter plot of PrimePCR™ ddPCR™ Mutation Detection Assay (FAM/HEX) targeting the *NOTCH1* c.3310G>A gene mutation in the tumor sample of patient RGR-56. The figure demonstrates the four clusters obtained with a mutant and wild type allele. In pink thresholds for channel 1 (FAM) and 2 (HEX) are shown.

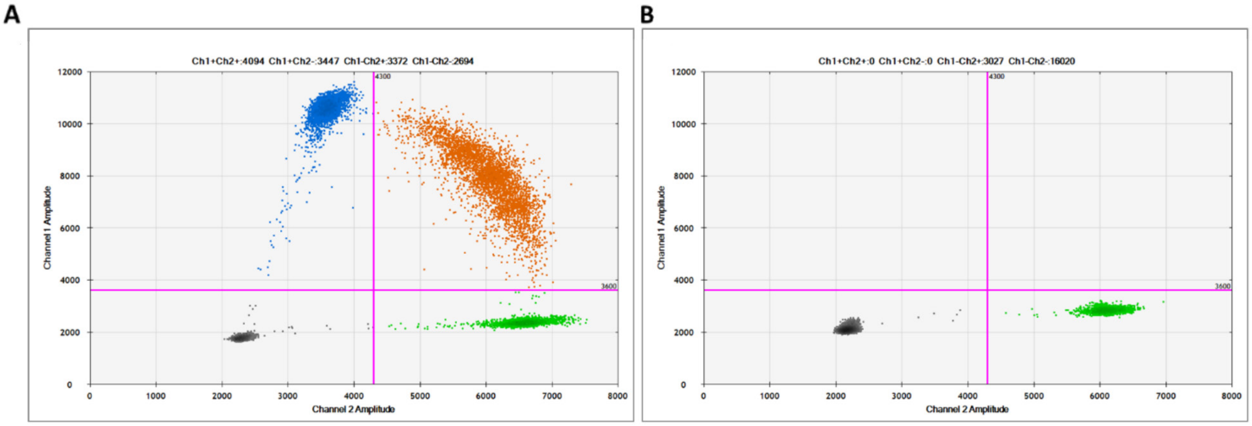

Supplementary Figure 3: Two-dimensional scatter plots of PrimePCR™ ddPCR™ Mutation Detection Assays (FAM/HEX) targeting the *TP53* c.710T>A gene mutation in patient RGR-58. (A) Plasma samples at progression and **(B)** in a wild type DNA whole blood sample, used as a negative control. The figure demonstrates the 4 clusters obtained with mutant (blue) and wild type (green) alleles. The droplets containing both alleles are shown in orange and grey droplets represents droplets without any template. In pink thresholds fixed for channel 1 (FAM) and 2 (HEX) according to optimization tests, are shown.

Supplementary Table 1: Baseline patient demographics and disease characteristics

	Population
**N patients**	20
**Age (years)**	66 (37-83)
**Sex**	
Men	12 (60%)
Women	8 (40%)
**Primary site of disease**	
Colon	15 (75%)
Rectum	3 (15%)
Colon and rectum	2 (10%)
**Prior therapies**	
Folfox	20 (100%)
Folfiri	20 (100%)
Capecitabine	8 (40%)
anti-EGFR	8 (40%)
anti-VEGF	14 (70%)
SIR-Spheres	1 (5%)
**Histology**	
Adenocarcinoma	20 (100%)
***KRAS* mutation**†	
No	10 (50%)
Yes	10 (50%)
**Sample used for analysis**	
Primary	18 (90%)
Metastatic	2 (10%)
**N samples analyzed**	112
Tumor tissue	20 (18%)
Whole blood	20 (18%)
Plasma	72 (64%)
**Median time between tissue and baseline plasma (days)**	1278 (220-3580)
**Median Regorafenib therapy (days)**	61 (13-400)

† *KRAS* mutation status was based on molecular record.

Supplementary Table 2: Results of targeted gene sequencing analysis on archived tumor samples and plasma samples at baseline and after 14 days (D14) of regorafenib therapy (cycle 1). VAF, variant allele frequency.

See Supplementary File 1

Supplementary Table 3: *KRAS* status comparison between PCR and targeted gene sequencing results

Patient	*KRAS* status according to:				
	External centres (original status)	Targeted sequencing analysis			
		Tumor	Plasma at baseline	Plasma at D14 (C1)	Blood
RGR-1	Wild Type	Mutated (27%) (codon 146)	Mutated	Mutated	Wild Type
RGR-2	Mutated	Mutated (19%)	Wild Type	Wild Type	Wild Type
RGR-4	Mutated	Mutated (7%)	Wild Type	Wild Type	Wild Type
RGR-7	Wild Type	Wild Type	Wild Type	Wild Type	Wild Type
RGR-14	Mutated	Mutated (37%)	Mutated	Mutated	Wild Type
RGR-24	Wild Type	Wild Type	Wild Type	Wild Type	Wild Type
RGR-26	Mutated	Mutated (29%)	Mutated	Mutated	Wild Type
RGR-28	Wild Type	Wild Type	Wild Type	Wild Type	Wild Type
RGR-30	Mutated	Wild Type	Wild Type	Wild Type	Wild Type
RGR-35	Mutated	Mutated (14%)	Mutated	Mutated	Wild Type
RGR-38	Mutated	Mutated (20%)	Mutated	Mutated	Wild Type
RGR-43	Wild Type	Wild Type	Wild Type	Wild Type	Wild Type
RGR-44	Wild Type	Wild Type	Mutated	Mutated	Wild Type
RGR-46	Wild Type	Mutated (35%)	Mutated	Wild Type	Wild Type
RGR-50	Mutated	Mutated (23%)	Mutated	Mutated	Wild Type
RGR-51	Mutated	Mutated (36%)	Mutated	Mutated	Wild Type
RGR-54	Mutated	Mutated (14%)	Mutated	Mutated	Wild Type
RGR-56	Wild Type	Wild Type	Wild Type	Wild Type	Wild Type
RGR-58	Wild Type	Mutated (1%)	Mutated	Mutated	Wild Type
RGR-61	Wild Type	Mutated (34%) (codon 61)	Mutated	Mutated	Wild Type

The grey lines represent a discordance between the PCR analysis in the external centers and targeted sequencing analysis. Patient RGR-1 and RGR-61 are not included in the discordant patients because targeted sequencing found a *KRAS* mutation on respectively codon 146 and 61 in their tumor. However, most centers only look for mutations on codons 12 and 13 as they represent approximately 95% of mutations in *KRAS* mutant CRC.

Supplemenary Table 4: Overview of the gene panel for targeted sequencing analysis

Panel A		Panel B	
**Gene classically mutated in CRC**		**Gene classically mutated in Cancer**	
ACVR1B	MSH6	ABL1	IDH2
ACVR2A	MTOR	AKT1	JAK2
AKT1	MUTYH	ALK	JAK3
APC	MYC	APC	KDR
ARID1A	NOTCH1	ATM	KIT
ATM	NRAS	BRAF	KRAS
AXIN2	PI3KCA	CDH1	MET
BMPR1A	PI3KR1	CDKN2A	MLH1
BRAF	PMS2	CSF1R	MPL
CTNNB1	PoID1	CTNNB1	NOTCH1
EGFR	PoIE	EGFR	NPM1
ERBB2	PTEN	ERBB2	NRAS
ERBB3	SLC9A9	ERBB4	PDGFRA
ERBB4	SMAD2	EZH2	PI3KCA
AMER1	SMAD3	FBXW7	PTEN
FBXW7	SMAD4	FGFR1	PTPN11
IGF1R	SOX9	FGFR2	RB1
IGF2	STK11	FGFR3	RET
IGF2R	TCF7L1	FLT3	SMAD4
KRAS	TCF7L2	GNA11	SMARCB1
MAP2K1	TGFBR1	GNAQ	SMO
MLH1	TGFBR2	GNAS	SRC
MSH2	TP53	HNF1A	STK11
	VEGF-A	HRAS	TP53
		IDH1	VHL

Supplemenary Table 5: Targeted gene variants and corresponding Biorad Prime PCR™ ddPCR™ Mutation Detection Assay references. Amplicon length and annealing temperature (Tm) is also shown for each assay. The grey marked custom designed assays were not functional.

See Supplementary File 2

Supplemenary Table 6: Thermal cycling conditions for Bio-Rad's T100 Touch Thermal Cycler

Cycling Step	T100™ Thermal Cycler				Settings
	Temperature (°C)	Time (min)	Ramp Rate	Repetitions	PCR volume (µL)
**Enzyme activation**	95	10:00		1	
**Denaturation**	94	00:30		40	
**Annealing/extension**	*52-58* *	01:10	~2°C/sec		40
**Enzyme deactivation**	98	10:00		1	
**Hold (optional)**	4	∞		-	

*Optimal annealing temperature used depending on Prime PCR™ ddPCR™ Mutation Detection Assay (see also Supplementary Table 5).

Supplemenary Table 7: The table lists the ddPCR events (mutant and wild type cases) obtained by analyzing archived tumor samples of 20 aCRC patients. Total number of copies per sample, Fractional Abundance (FA %) and Poisson CI. (95%) are shown.

See Supplementary File 3

Supplemenary Table 8: The tables list the ddPCR events (mutant and wild type cases) obtained by analyzing serial plasma samples at different time points (baseline until progression) of 20 aCRC patients. Total number of copies per sample, Fractional Abundance (FA %) and Poisson CI. (95%) are shown.

See Supplementary File 4
