# Supplementary material for: Circulating tumor DNA in early response assessment and monitoring of advanced colorectal cancer treated with a multi-kinase inhibitor
Source: Oncotarget. 2018 Apr 3;9(25):17756–69. doi: 10.18632/oncotarget.24879 (PMC5915153; doi:10.18632/oncotarget.24879)
Supplement: Supplementary file 2 [file oncotarget-09-17756-s002.docx]

| **Mutated genes** | **DNA variant** | **Tumor** | | **Plasma at baseline** | | **Plasma at C1** | |
| --- | --- | --- | --- | --- | --- | --- | --- |
|  |  | VAF* | Coverage | VAF* | Coverage | VAF* | Coverage |
| **RGR-1** |  | Day -756 | | Day 0 | | Day 14 | |
| MSH2 | c.2590G>A^1^ | 12,33% | 462 |  |  |  |  |
| APC | c.637C>T² | 16,83% | 493 | 26,13% | 29997 | 16,55% | 29122 |
| APC | c.2098_2099delAA² | 34,67% | 1024 | 41,60% | 24403 | 18,54% | 22091 |
| TCF7L2 | c.85G>T² | 22,45% | 2241 | 46,02% | 18419 | 18,24% | 22699 |
| KRAS | c.437C>T² | 26,84% | 2355 | 57,47% | 7371 | 20,72% | 7549 |
| TP53 | c.524G>A² | 34,94% | 1577 | 67,46% | 36087 | 28,94% | 27696 |
| **RGR-2** |  | Day -2201 | | Day 0 | | Day 14 | |
| MSH2 | c.49G>A^1^ | 57,25% | 138 |  |  |  |  |
| FBXW7 | c.1397C>T | 11,80% | 339 | <1% | 105003 | <1% | 104545 |
| APC | c.4128T>A² | 36,55% | 145 | 2,05% | 58880 | <1% | 53738 |
| MYC | c.151C>T² | 12,58% | 310 | <1% | 5420 | <1% | 3295 |
| POLE | c.3958C>T² | 22,86% | 210 | <1% | 61167 | <1% | 38935 |
| ERBB2 | c.59_60insG² | 44,69% | 461 | <1% | 88361 | <1% | 91393 |
| KRAS | c.35G>T² | 18,97% | 58 |  |  |  |  |
| **RGR-4** |  | Day -398 | | Day 0 | | Day 14 | |
| PIK3CA | c.3140A>G² | 36,24% | 3193 | 36,18% | 24718 | 23,55% | 28022 |
| FBXW7 | c.859C>T² | 18,25% | 2016 |  |  |  |  |
| APC | c.2995C>T² | 24,73% | 550 |  |  |  |  |
| BRAF | c.1799T>A² | 19,51% | 1107 | 21,12% | 14258 | 9,95% | 15634 |
| TP53 | c.659A>G² | 55,05% | 1873 | 47,38% | 55007 | 23,10% | 82744 |
| AMER1 | c.1756C>T² | 52,59% | 829 | 43,81% | 41198 | 28,00% | 33928 |
| KRAS | c.35G>A² | 7,14% | 1023 |  |  |  |  |
| **RGR-7** |  | Day -1266 | | Day 0 | | Day 14 | |
| PIK3R1 | c.305C>A² | 19,08% | 1321 | 44,53% | 145149 | 49,27% | 63626 |
| TP53 | c.641A>G³ | 24,16% | 1337 | 42,55% | 55283 | 30,60% | 50616 |
| **RGR-14** |  | Day -1552 | | Day 0 | | Day 14 | |
| KRAS | c.35G>A² | 36,62% | 822 | 24,69% | 25419 | 10,05% | 25549 |
| **RGR-24** |  | Day -3528 | | Day 0 | | Day 14 | |
| PIK3CA | c.2144T>C^1^ | 21,05% | 817 | <1% | 187229 | <1% | 172179 |
| APC | c.3095C>A² | 18,73% | 758 | 54,17% | 42621 | 12,58% | 78237 |
| **RGR-26** |  | Day -909 | | Day 0 | | Day 14 | |
| MSH2 | c.418G>A^1^ | 15,33% | 646 |  |  |  |  |
| KRAS | c.34G>A² | 28,77% | 146 | 15,99% | 33837 | 8,61% | 25266 |
| TP53 | c.586C>T² | 37,50% | 264 | 20,32% | 48502 | 12,35% | 40005 |
| **RGR-28** |  | Day -2858 | | Day 0 | | Day 14 | |
| SMAD3 | c.1029C>A^1^ | 21,19% | 859 | 5,72% | 59738 | <1% | 73219 |
| TP53 | c.818G>A³ | 34,50% | 626 | 5,36% | 43822 | <1% | 94549 |
| **RGR-30** |  | Day -1773 | | Day 0 | | Day 14 | |
| NRAS | c.35G>T² | 51,04% | 2782 | 42,93% | 26938 | 26,00% | 31798 |
| ATM | c.4060C>T^5^ | 24,91% | 281 | <1% | 42936 | <1% | 81820 |
| TP53 | c.733G>A^1^ | 51,35% | 3447 | 38,50% | 33127 | 15,61% | 45359 |
| SMAD2 | c.794C>T^4^ | 21,89% | 201 | <1% | 19916 | <1% | 57077 |
| MSH2 | c.1315C>T^1^ | 20,15% | 412 | <1% | 47214 | <1% | 90647 |
| CTNNB1 | c.937-1G>A^6^ | 20,28% | 355 | ** | ** | ** | ** |
| PIK3CA | c.1091G>A^5^ | 23,24% | 185 | <1% | 35781 | <1% | 61455 |
| PIK3R1 | c.34G>A^5^ | 20,47% | 342 | <1% | 45611 | <1% | 88433 |
| APC | c.2054G>A² | 42,01% | 3216 | 32,34% | 154878 | 19,63% | 202205 |
| APC | c.2000_2004delGAGCG² | 30,67% | 150 | 25,89% | 9560 | 10,51% | 11125 |
| **RGR-35** |  | Day -1545 | | Day 0 | | Day 14 | |
| ERBB4 | c.3521G>A^5^ | 11,48% | 5714 | 6,26% | 157322 | 1,62% | 176640 |
| PIK3CA | c.1633G>A² | 12,74% | 2362 | 9,93% | 49096 | 1,49% | 108217 |
| APC | c.2093T>A² | 12,08% | 2541 | 8,37% | 120389 | 1,20% | 134419 |
| APC | c.2532_2535delGATG² | 13,63% | 1746 | 7,00% | 231505 | ** | ** |
| KRAS | c.34G>T² | 13,21% | 1052 | 7,90% | 27900 | <1% | 18506 |
| TP53 | c.743G>A³ | 19,51% | 1712 | 9,27% | 39263 | 1,13% | 163131 |
| **RGR-38** |  | Day -733 | | Day 0 | | Day 14 | |
| APC | c.2821G>T² | 21,28% | 1725 | 5,03% | 111528 | 1,54% | 96683 |
| KRAS | c.35G>A² | 19,94% | 1073 | 2,78% | 55243 | 1,64% | 36192 |
| **RGR-43** |  | Day -1270 | | Day 0 | | Day 14 | |
| NRAS | c.35G>A² | 26,49% | 1295 | 4,34% | 33272 | <1% | 44985 |
| APC | c.847C>T² | 18,03% | 183 | 5,95% | 46176 | <1% | 31222 |
| MAP2K1 | c.383G>A^1^ | 33,91% | 1398 | 10,39% | 331971 | <1% | 399049 |
| TP53 | c.743G>A³ | 37,72% | 1853 | 7,11% | 53589 | <1% | 287822 |
| **RGR-44** |  | Day -1598 | | Day 0 | | Day 14 | |
| PIK3CA | c.247TTT>AAA^1^ | 31,23% | 1489 | 36,50% | 66484 | 30,04% | 22477 |
| APC | c.694C>T² | 12,57% | 2402 | 1,50% | 140513 | 1,46% | 48179 |
| APC | c.3944C>G² | 8,26% | 230 | 17,53% | 5447 | 11,16% | 2813 |
| KRAS | c.183A>C² |  |  | 12,54% | 40172 | 10,40% | 27249 |
| **RGR-46** |  | Day -616 | | Day 0 | | Day 14 | |
| PIK3CA | c.1633G>A² | 39,22% | 1986 | 13,91% | 56793 | 8,48% | 52506 |
| APC | c.2626C>T² | 17,12% | 3020 | 8,98% | 49757 | 4,51% | 133232 |
| APC | c.4285C>T² | 34,28% | 1730 | 10,15% | 49077 | 5,72% | 42563 |
| KRAS | c.37G>C² | 35,04% | 508 | 7,66% | 25286 | 5,00% | 11181 |
| TP53 | c.864T>G^1^ | 50,54% | 1292 | 20,87% | 47601 | 14,72% | 40844 |
| RET | c.2751G>A |  |  | 9,24% | 26129 | 5,07% | 15423 |
| **RGR-50** |  | Day -803 | | Day 0 | | Day 14 | |
| PIK3CA | c.1624G>A² | 20,42% | 3207 | 31,06% | 48520 | 16,06% | 34539 |
| KRAS | c.35G>T² | 22,79% | 1009 | 54,82% | 40825 | 29,03% | 22029 |
| TP53 | c.329G>C^1^ | 24,14% | 1914 | 39,57% | 40796 | 19,40% | 39077 |
| SMAD4 | c.388C>T³ | 20,45% | 572 | 33,26% | 52303 | 14,71% | 30425 |
| **RGR-51** |  | Day -233 | | Day 0 | | Day 14 | |
| ACVR2A | c.798C>G^1^ | 31,71% | 1479 | 30,26% | 49708 | 18,30% | 86021 |
| ERBB4 | c.1775A>T^5^ | 27,24% | 2533 | 22,56% | 29541 | 13,89% | 42389 |
| APC | c.1495C>T² | 33,40% | 1581 | 20,69% | 49397 | 13,33% | 101006 |
| APC | c.4285C>T² | 35,43% | 2094 | 24,32% | 32475 | 15,41% | 39952 |
| PTEN | c.138_139insG² | 13,94% | 1184 | 13,54% | 58222 | 7,77% | 98882 |
| PTEN | c.697C>T² | 32,12% | 1535 | 22,71% | 36789 | 13,86% | 51238 |
| KRAS | c.35G>C² | 35,34% | 1149 | 25,84% | 16793 | 16,99% | 26927 |
| TP53 | c.733G>A^4^ | 47,14% | 1610 | 36,86% | 29484 | 21,07% | 44489 |
| SMAD4 | c.1544G>C² | 1,06% | 1224 | 17,90% | 14644 | 7,24% | 30590 |
| **RGR-54** |  | Day -1357 | | Day 0 | | Day 14 | |
| PIK3CA | c.3145G>C^5^ | 13,48% | 3116 | <1% | 16376 | <1% | 75179 |
| APC | c.694C>T² | 20,13% | 2146 | <1% | 46455 | <1% | 206524 |
| TCF7L2 | c.3G>A³ | 14,96% | 1558 | <1% | 16058 | <1% | 70156 |
| KRAS | c.38G>A² | 12,36% | 1084 | <1% | 19523 | <1% | 79239 |
| TP53 | c.637C>T² | 22,42% | 1699 | <1% | 82150 | <1% | 215723 |
| **RGR-56** |  | Day -3558 | | Day 0 | | Day 14 | |
| APC | c.4099C>T² | 27,06% | 473 | 19,68% | 33616 | 7,54% | 35597 |
| NOTCH1 | c.3310G>A^1^ | 31,09% | 431 | 16,87% | 86916 | 7,90% | 43326 |
| TP53 | c.1009C>T^5^ | 41,40% | 831 | 39,92% | 51730 | 18,22% | 44956 |
| **RGR-58** |  | Day -360 | | Day 0 | | Day 14 | |
| APC | c.2169_2170insC² |  |  | 43,07% | 19486 | 12,12% | 21313 |
| KRAS | c.34G>T² | 2,86% | 1234 | 63,42% | 25547 | 20,94% | 24727 |
| TP53 | c.710T>A² | 1,47% | 679 | 64,11% | 27471 | 19,59% | 29412 |
| **RGR-61** |  | Day -220 | | Day 0 | | Day 14 | |
| FBXW7 | c.1262delC | 61,42% | 3087 | 46,72% | 87471 | 31,50% | 57054 |
| APC | c.2510_2511insT² | 30,85% | 1802 | 20,00% |  | 20,00% |  |
| KRAS | c.183A>C² | 33,36% | 2908 | 23,27% | 29628 | 32,90% | 39807 |
| TP53 | c.473G>A^5^ | 63,70% | 887 | 27,62% | 22335 | 29,85% | 62044 |
| SOX9 | c.696delC² | 60,49% | 329 |  |  |  |  |
|  |  |  |  |  |  |  |  |
| ^1^ | Unknown |  |  |  | *Variant Allele frequency | |  |
| ^2^ | Damaging |  |  |  | **not evaluable by software | |  |
| ^3^ | Potentially damaging | |  |  |  |  |  |
| ^4^ | Probably polymorphism | |  |  | New mutation in plasmas | |  |
| ^5^ | Uknown probably benign | |  |  |  |  |  |
| ^6^ | Splice variant |  |  |  |  |  |  |

**Table S2.** Results of targeted gene sequencing analysis on archived tumor samples and plasma samples at baseline and after 14 days (D14) of regorafenib therapy (cycle 1). VAF, variant allele frequency.
