# Supplementary material for: Circulating tumor DNA in early response assessment and monitoring of advanced colorectal cancer treated with a multi-kinase inhibitor
Source: Oncotarget. 2018 Apr 3;9(25):17756–69. doi: 10.18632/oncotarget.24879 (PMC5915153; doi:10.18632/oncotarget.24879)
Supplement: Supplementary file 3 [file oncotarget-09-17756-s003.docx]

| **Gene** | **Mutation** | **Variant** | **Mutation Assay** | **Reference Assay** | **Amplicon length** | **Tm (°C)** |
| --- | --- | --- | --- | --- | --- | --- |
| **APC** | p.R213* | c.637C>T | dHsaIS2505714 | dHsaIS2505715 | 69 | 52 |
| **APC** | p.Q1429* | c.4285C>T | dHsaIS2505378 | dHsaIS2505379 | 70 | 52 |
| **APC** | p.Q1367* | c.4099C>T | dHsaIS2501856 | dHsaIS2501857 | 62 | 52 |
| **APC** | p.R283* | c.847C>T | dHsaIS2501652 | dHsaIS2501653 | 70 | 55 |
| **APC** | p.R232* | c.694C>T | dHsaIS2501386 | dHsaIS2501387 | 70 | 52 |
| **APC** | p.Y1376* | c.4128T>A | dHsaIS2501112 | dHsaIS2501113 | 65 | 55 |
| **BRAF** | p.V600E | c.1799T>A | dHsaCP2000027 | dHsaCP2000028 | 91 | 55 |
| **KRAS** | p.G13R | c.37G>C | dHsaCP2506874 | dHsaCP2506875 | 59 | 55 |
| **KRAS** | p.G12D | c.35G>A | dHsaCP2500596 | dHsaCP2500597 | 57 | 55 |
| **KRAS** | p.G12C | c.34G>T | dHsaCP2500584 | dHsaCP2500585 | 57 | 55 |
| **KRAS** | p.G12A | c.35G>C | dHsaCP2500586 | dHsaCP2500587 | 57 | 55 |
| **KRAS** | p.Q61H | c.183A>C | dHsaCP2500578 | dHsaCP2500579 | 65 | 55 |
| **KRAS** | p.G12S | c.34G>A | dHsaCP2500588 | dHsaCP2500589 | 57 | 55 |
| **KRAS** | p.G12V | c.35G>T | dHsaCP2500592 | dHsaCP2500593 | 57 | 52 |
| **KRAS** | p.G13D | c.38G>A | dHsaCP2500598 | dHsaCP2500599 | 57 | 55 |
| **NRAS** | p.G12V | c.35G>T | dHsaCP2500528 | dHsaCP2500529 | 57 | 55 |
| **NRAS** | p.G12D | c.35G>A | dHsaCP2000095 | dHsaCP2000096 | 70 | 55 |
| **PIK3CA** | p.G1049R | c.3145G>C | dHsaCP2500574 | dHsaCP2500575 | 74 | 52 |
| **PIK3CA** | p.H1047R | c.3140A>G | dHsaCP2000077 | dHsaCP2000078 | 80 | 58 |
| **PIK3CA** | p.E545K | c.1633G>A | dHsaCP2000075 | dHsaCP2000076 | 78 | 55 |
| **PIK3CA** | p.E542K | c.1624G>A | dHsaCP2000073 | dHsaCP2000074 | 78 | 55 |
| **TP53** | p.R337C | c.1009C>T | dHsaIS2505928 | dHsaIS2505929 | 79 | 55 |
| **TP53** | p.M237K | c.710T>A | dHsaIS2503142 | dHsaIS2503143 | 64 | 55 |
| **TP53** | p.R110P | c.329G>C | dHsaIS2501294 | dHsaIS2501295 | 65 | 52 |
| **TP53** | p.H214R | c.641A>G | dHsaIS2500824 | dHsaIS2500825 | 64 | 55 |
| **TP53** | p.G245S | c.733G>A | dHsaCP2506746 | dHsaCP2506747 | 65 | 52 |
| **TP53** | p.R158H | c.473G>A | dHsaCP2500522 | dHsaCP2500523 | 62 | 52 |
| **TP53** | p.R248Q | c.743G>A | dHsaCP2000127 | dHsaCP2000128 | 62 | 58 |
| **TP53** | p.R196* | c.586C>T | dHsaCP2000121 | dHsaCP2000122 | 65 | 52 |
| **TP53** | p.R273H | c.818G>A | dHsaCP2000109 | dHsaCP2000110 | 65 | 52 |
| **TP53** | p.R175H | c.524G>A | dHsaCP2000105 | dHsaCP2000106 | 65 | 52 |
| **APC** | p.S1032* | c.3095C>A | **Custom Designed Assays from Bio-Rad following design guidelines apply to ddPCR primer** | | | 55 |
| **APC** | p.W685* | c.2054G>A |  |  |  | 55 |
| **APC** | p.E941* | c.2821G>T |  |  |  | 55 |
| **APC** | p.V1377Rfs*1385 | c.2169_2170insC |  |  |  | - |
| **APC** | p.A1492Cfs*1513 | c.2510_2511insT |  |  |  | 55 |
| **FBXW7** | p.A422Qfs*443 | c.1262delC |  |  |  | 58 |
| **ERBB2** | p.R429Efs*441;p.R459Efs*471 | c.59_60insG |  |  |  | - |
| **KRAS** | p.A146V | c.437C>T |  |  |  | 55 |
| **NOTCH1** | p.A1104T | c.3310G>A |  |  |  | 52 |
| **PIK3CA** | p.L715S | c.2144T>C |  |  |  | - |
| **PIK3CA** | p.G364E | c.1091G>A |  |  |  | - |
| **PIK3CA** | p.F83L | c.247TTT>AAA |  |  |  | 52 |
| **PIK3R1** | p.S102* | c.305C>A |  |  |  | 52 |
| **SMAD3** | p.F343L | c.1029C>A |  |  |  | 52 |
| **TP53** | p.N288K | c.864T>G |  |  |  | - |

**Table S5. Targeted gene variants and corresponding Biorad Prime PCR^TM^ ddPCR^TM^ Mutation Detection Assay references.** Amplicon length and annealing temperature (Tm) is also shown for each assay. The grey marked custom designed assays were not functional.
