# Supplementary material for: Circulating tumor DNA in early response assessment and monitoring of advanced colorectal cancer treated with a multi-kinase inhibitor
Source: Oncotarget. 2018 Apr 3;9(25):17756–69. doi: 10.18632/oncotarget.24879 (PMC5915153; doi:10.18632/oncotarget.24879)
Supplement: Supplementary file 4 [file oncotarget-09-17756-s004.docx]

|  |  |  | **Tumor** | | | | | | |
| --- | --- | --- | --- | --- | --- | --- | --- | --- | --- |
| Gene | Mutation | Patient | MUT events | WT events | MUT copies/20µL well | WT copies/20µL well | TOT copies/ well | Fractional Abundance (FA %) | Poisson 95% C.I |
| **APC** | p.R213* | 1 | 455 | 3117 | 902 | 7020 | 7922 | 11,4 | 12,4-10,4 |
| **APC** | p.Q1429* | 46 | 8989 | 14904 | 15400 | 37440 | 52840 | 29,1 | 29,7-28,6 |
|  |  | 51 | 3275 | 6526 | 4980 | 11240 | 16220 | 30,7 | 31,6-29,8 |
| **APC** | p.Q1367* | 56 | 3871 | 6996 | 8960 | 20000 | 28960 | 31 | 31,8-30,1 |
| **APC** | p.R283* | 43 | 7138 | 11375 | 14780 | 32020 | 46800 | 31,6 | 32,3-30,9 |
| **APC** | p.R232* | 44 | 5039 | 14416 | 8620 | 49320 | 57940 | 14,87 | 15,3-14,4 |
|  |  | 54 | 1089 | 4125 | 1720 | 7320 | 9040 | 19 | 20,1-18 |
| **APC** | p.Y1376* | 2 | 4392 | 2884 | 9720 | 5920 | 15640 | 62,2 | 63,3-61,1 |
| **APC** | p.S1032* | 24 | 2167 | 5032 | 3760 | 9880 | 13640 | 27,6 | 28,6-26,6 |
| **APC** | p.W685* | 30 | 2335 | 5473 | 4180 | 11340 | 15520 | 27 | 27,9-26 |
| **APC** | p.E941* | 38 | 1125 | 3490 | 1880 | 6440 | 8320 | 22,7 | 23,9-21,5 |
| **APC** | p.A1492Cfs*1513 | 61 | 5851 | 8656 | 13520 | 24500 | 38020 | 35,6 | 36,4-34,8 |
| **BRAF** | p.V600E | 4 | 2591 | 742 | 5200 | 18500 | 23700 | 21,9 | 22,7-21,2 |
| **FBXW7** | p.A422Qfs*443 | 61 | 12266 | 9296 | 25540 | 16400 | 41940 | 60,9 | 61,9-60,2 |
| **KRAS** | p.G13R | 46 | 6987 | 11183 | 16100 | 37040 | 53140 | 30,3 | 31-29,6 |
| **KRAS** | p.G12D | 4 | 843 | 8044 | 1298 | 16900 | 18198 | 7,14 | 7,61-6,67 |
|  |  | 14 | 8930 | 12140 | 19000 | 32900 | 51900 | 36,6 | 37,3-35,9 |
|  |  | 38 | 7524 | 14272 | 14460 | 48160 | 62620 | 23,1 | 23,6-22,6 |
| **KRAS** | p.G12C | 35 | 1039 | 5670 | 1604 | 10480 | 12084 | 13,3 | 14-12,5 |
|  |  | 58 | 56 | 1383 | 94 | 2440 | 2534 | 3,7 | 4,7-2,8 |
| **KRAS** | p.G12A | 51 | 1832 | 3519 | 4080 | 8600 | 12680 | 52,2 | 33,4-31 |
| **KRAS** | p.Q61H | 44 | 0 | 11494 | 0 | 80000 | 80000 | - | - |
|  |  | 61 | 4862 | 7499 | 8780 | 15400 | 24180 | 36,3 | 37,2-35,5 |
| **KRAS** | p.G12S | 26 | 1478 | 4114 | 1948 | 5880 | 7828 | 24,9 | 26-23,8 |
| **KRAS** | p.G12V | 2 | 1854 | 4070 | 2980 | 7100 | 10080 | 29,5 | 30,7-28,4 |
|  |  | 50 | 369 | 1211 | 1500 | 5300 | 6800 | 22 | 24-20 |
| **KRAS** | p.G13D | 54 | 905 | 3550 | 2260 | 10440 | 12700 | 17,8 | 18,8-16,7 |
| **KRAS** | p.A146V | 1 | 794 | 2128 | 1500 | 4280 | 5780 | 26 | 27,6-24,5 |
| **NOTCH1** | p.A1104T | 56 | 4623 | 8377 | 12500 | 32300 | 44800 | 27,9 | 28,6-27,1 |
| **NRAS** | p.G12V | 30 | 5086 | 5257 | 14260 | 14920 | 29180 | 48,9 | 49,8-47,9 |
| **NRAS** | p.G12D | 43 | 6009 | 10780 | 13960 | 38260 | 52220 | 26,8 | 27,4-26,1 |
| **PIK3CA** | p.G1049R | 54 | 1055 | 4903 | 1800 | 9920 | 11720 | 15,4 | 16,3-14,6 |
| **PIK3CA** | p.H1047R | 4 | 5213 | 9610 | 7820 | 17320 | 25140 | 31,1 | 31,8-30,4 |
| **PIK3CA** | p.E545K | 35 | 1337 | 6079 | 1960 | 10600 | 12560 | 15,6 | 16,4-14,8 |
|  |  | 46 | 3566 | 5549 | 11040 | 20580 | 31620 | 34,9 | 35,1-33,8 |
| **PIK3CA** | p.E542K | 50 | 404 | 1527 | 784 | 3120 | 3904 | 20,1 | 21,9-18,4 |
| **PIK3CA** | p.F83L | 44 | 7180 | 10163 | 20100 | 39480 | 59580 | 37,7 | 34,5-33 |
| **PIK3R1** | p.S102* | 7 | 3278 | 8172 | 7000 | 24120 | 31120 | 22,5 | 23,2-21,8 |
| **SMAD3** | p.F343L | 28 | 3234 | 10564 | 5040 | 23340 | 28380 | 17,7 | 18,3-17,2 |
| **TP53** | p.R337C | 56 | 6156 | 8299 | 11780 | 17820 | 29600 | 39,8 | 40,6-39 |
| **TP53** | p.M237K | 58 | 79 | 5021 | 172 | 14700 | 14872 | 1,16 | 1,42-0,9 |
| **TP53** | p.R110P | 50 | 730 | 1778 | 1150 | 2900 | 4050 | 28,4 | 30,1-26,6 |
| **TP53** | p.H214R | 7 | 3446 | 9741 | 7300 | 32940 | 40240 | 18,1 | 18,7-17,5 |
| **TP53** | p.G245S | 51 | 2088 | 2106 | 4580 | 4620 | 9200 | 49,8 | 51,3-48,2 |
|  |  | 30 | 3835 | 3906 | 9660 | 9880 | 19540 | 49,4 | 50,6-48,3 |
| **TP53** | p.R158H | 61 | 11282 | 7876 | 43840 | 20960 | 64800 | 67,6 | 68,3-67 |
| **TP53** | p.R175H | 1 | 1278 | 2432 | 2760 | 5580 | 8340 | 33,2 | 34,7-31,6 |
| **TP53** | p.R248Q | 35 | 1409 | 5938 | 1860 | 9080 | 10940 | 17 | 17,8-16,2 |
|  |  | 43 | 8623 | 11975 | 16320 | 27900 | 44220 | 36,9 | 37,6-36,2 |
| **TP53** | p.R196* | 26 | 4536 | 9427 | 6880 | 17600 | 24480 | 28,1 | 28,8-27,4 |
| **TP53** | p.R273H | 28 | 5530 | 5978 | 11240 | 12440 | 23680 | 47,5 | 48,4-46,6 |

**Table S7.** The table lists the ddPCR events (mutant and wild type cases) obtained by analyzing archived tumor samples of 20 aCRC patients. Total number of copies per sample, Fractional Abundance (FA %) and Poisson CI. (95%) are shown.
