# Supplementary material for: Circulating tumor DNA in early response assessment and monitoring of advanced colorectal cancer treated with a multi-kinase inhibitor
Source: Oncotarget. 2018 Apr 3;9(25):17756–69. doi: 10.18632/oncotarget.24879 (PMC5915153; doi:10.18632/oncotarget.24879)
Supplement: Supplementary file 5 [file oncotarget-09-17756-s005.docx]

|  |  |  | **Plasma at baseline** | | | | | | |
| --- | --- | --- | --- | --- | --- | --- | --- | --- | --- |
| Gene | Mutation | Patient | MUT events | WT events | MUT copies/20µL well | WT copies/20µL well | TOT copies/ well | Fractional Abundance (FA %) | Poisson 95% C.I |
| **APC** | p.R213* | 1 | 4166 | 9618 | 5540 | 15560 | 21100 | 26,2 | 26,9-25,5 |
| **APC** | p.Q1429* | 46 | 273 | 1498 | 450 | 2580 | 3030 | 14,8 | 16,5-13,2 |
|  |  | 51 | 3468 | 7281 | 4240 | 9980 | 14220 | 29,8 | 30,6-28,9 |
| **APC** | p.Q1367* | 56 | 215 | 472 | 410 | 910 | 1320 | 31,1 | 34,5-27,6 |
| **APC** | p.R283* | 43 | 125 | 1572 | 202 | 2680 | 2882 | 7 | 8,2-5,8 |
| **APC** | p.R232* | 44 | 26 | 1853 | 36 | 2700 | 2736 | 1,3 | 1,8-0,8 |
|  |  | 54 | 1 | 1707 | 1,4 | 2460 | 2461,4 | 0,06 | 0,19-0 |
| **APC** | p.Y1376* | 2 | 24 | 571 | 38 | 922 | 960 | 4 | 5,5-2,4 |
| **APC** | p.S1032* | 24 | 2136 | 2900 | 6340 | 5080 | 11420 | 41,7 | 43-40,3 |
| **APC** | p.W685* | 30 | 2054 | 4115 | 2620 | 5580 | 8200 | 32 | 33,1-30,8 |
| **APC** | p.E941* | 38 | 85 | 925 | 138 | 1536 | 1674 | 8,2 | 9,9-6,5 |
| **APC** | p.A1492Cfs*1513 | 61 | 623 | 1836 | 994 | 3060 | 4054 | 24,5 | 26,2-22,8 |
| **BRAF** | p.V600E | 4 | 2348 | 6297 | 4000 | 12780 | 16780 | 23,8 | 24,7-23 |
| **FBXW7** | p.A422Qfs*443 | 61 | 1384 | 2573 | 1646 | 3160 | 4806 | 34,3 | 35,7-32,8 |
| **KRAS** | p.G13R | 46 | 172 | 1220 | 274 | 2020 | 2294 | 11,9 | 13,6-10,3 |
| **KRAS** | p.G12D | 4 | 3 | 5133 | 5 | 10840 | 10845 | 0,05 | 0,1-0 |
|  |  | 14 | 1240 | 2591 | 1738 | 3800 | 5538 | 31,4 | 32,9-30 |
|  |  | 38 | 102 | 1141 | 138 | 1590 | 1728 | 8 | 9,5-6,5 |
| **KRAS** | p.G12C | 35 | 91 | 1002 | 142 | 1604 | 1746 | 8,1 | 9,7-6,5 |
|  |  | 58 | 4655 | 3086 | 7540 | 4720 | 12260 | 61,5 | 62,6-60,4 |
| **KRAS** | p.G12A | 51 | 2113 | 4696 | 3120 | 7620 | 10740 | 29,1 | 30,2-28 |
| **KRAS** | p.Q61H | 44 | 2189 | 10507 | 3280 | 23160 | 26440 | 12,4 | 12,9-11,9 |
|  |  | 61 | 1207 | 2475 | 1860 | 3980 | 5840 | 31,8 | 33,3-30,3 |
| **KRAS** | p.G12S | 26 | 254 | 1035 | 366 | 1524 | 1890 | 19,3 | 21,5-17,2 |
| **KRAS** | p.G12V | 2 | 8 | 580 | 10,8 | 792 | 802,8 | 1,3 | 2,3-0,4 |
|  |  | 50 | 3364 | 2260 | 6080 | 3920 | 10000 | 60,8 | 62,1-59,5 |
| **KRAS** | p.G13D | 54 | 4 | 1332 | 6,2 | 2180 | 2186,2 | 0,29 | 0,58-0 |
| **KRAS** | p.A146V | 1 | 6375 | 4778 | 13160 | 9120 | 22280 | 59,1 | 60-58,2 |
| **NOTCH1** | p.A1104T | 56 | 111 | 506 | 184 | 852 | 1036 | 17,8 | 20,8-14,8 |
| **NRAS** | p.G12V | 30 | 3065 | 3585 | 5220 | 6240 | 11460 | 45,6 | 46,8-44,4 |
| **NRAS** | p.G12D | 43 | 142 | 1752 | 226 | 2960 | 3186 | 7,1 | 8,2-6 |
| **PIK3CA** | p.G1049R | 54 | 0 | 1114 | 0 | 2040 | 2040 | 0 | - |
| **PIK3CA** | p.H1047R | 4 | 2523 | 3525 | 3020 | 4340 | 7360 | 41,1 | 42,3-39,8 |
| **PIK3CA** | p.E545K | 35 | 140 | 1506 | 192 | 2140 | 2332 | 8,2 | 9,5-6,9 |
|  |  | 46 | 970 | 4885 | 1920 | 11880 | 13800 | 14 | 15,1-13,8 |
| **PIK3CA** | p.E542K | 50 | 1172 | 2365 | 2140 | 4520 | 6660 | 32 | 33,6-30,5 |
| **PIK3CA** | p.F83L | 44 | 1218 | 1697 | 2020 | 2860 | 4880 | 41,4 | 43,1-39,6 |
| **PIK3R1** | p.S102* | 7 | - | - | - | - | - | - | - |
| **SMAD3** | p.F343L | 28 | 112 | 1414 | 180 | 2380 | 2560 | 7 | 8,3-5,8 |
| **TP53** | p.R337C | 56 | 338 | 417 | 488 | 604 | 1092 | 44,7 | 48,3-41,2 |
| **TP53** | p.M237K | 58 | 5207 | 3284 | 10300 | 5960 | 16260 | 63,4 | 64,4-62,3 |
| **TP53** | p.R110P | 50 | 2158 | 2740 | 3000 | 3880 | 6880 | 43,6 | 45-42,2 |
| **TP53** | p.H214R | 7 | - | - | - | - | - | - | - |
| **TP53** | p.G245S | 51 | 2302 | 2966 | 4160 | 5520 | 9680 | 43 | 44,4-41,7 |
|  |  | 30 | 1475 | 1846 | 3040 | 3860 | 6900 | 44 | 45,7-42,3 |
| **TP53** | p.R158H | 61 | 1067 | 1900 | 1740 | 3180 | 4920 | 35,3 | 37-33,6 |
| **TP53** | p.R175H | 1 | 6950 | 3221 | 13760 | 5400 | 19160 | 71,8 | 72,6-70,9 |
| **TP53** | p.R248Q | 35 | 203 | 1089 | 246 | 2680 | 2926 | 8,5 | 9,6-7,3 |
|  |  | 43 | 195 | 2492 | 224 | 3040 | 3264 | 6,9 | 7,8-5,9 |
| **TP53** | p.R196* | 26 | 604 | 1920 | 776 | 2560 | 3336 | 23,3 | 24,9-21,6 |
| **TP53** | p.R273H | 28 | 152 | 1192 | 222 | 1800 | 2022 | 11 | 12,6-9,3 |

|  |  |  | **Plasma at D14 (C1)** | | | | | | |
| --- | --- | --- | --- | --- | --- | --- | --- | --- | --- |
| Gene | Mutation | Patient | MUT events | WT events | MUT copies/20µL well | WT copies/20µL well | TOT copies/ well | Fractional Abundance (FA %) | Poisson 95% C.I |
| **APC** | p.R213* | 1 | 2015 | 10827 | 2580 | 19200 | 21780 | 11,84 | 12,34-11,34 |
| **APC** | p.Q1429* | 46 | 1685 | 12378 | 2680 | 36680 | 39360 | 6,8 | 7,12-6,47 |
|  |  | 51 | 6603 | 16672 | 9120 | 39200 | 48320 | 18,87 | 19,32-18,41 |
| **APC** | p.Q1367* | 56 | 1516 | 7538 | 2400 | 15540 | 17940 | 13,4 | 14,1-12,8 |
| **APC** | p.R283* | 43 | 12 | 6372 | 18 | 14,2 | 32,2 | 0,15 | 0,23-0,006 |
| **APC** | p.R232* | 44 | 83 | 5766 | 106 | 8900 | 9006 | 1,19 | 1,4-0,93 |
|  |  | 54 | 8 | 6568 | 10,2 | 10220 | 10230,2 | 0,1 | 0,17-0,003 |
| **APC** | p.Y1376* | 2 | 0 | 1260 | 0 | 2020 | 2020 | 0 | - |
| **APC** | p.S1032* | 24 | 1461 | 5328 | 2300 | 9760 | 12060 | 19,1 | 20-18,2 |
| **APC** | p.W685* | 30 | 1000 | 3900 | 1446 | 6220 | 7666 | 18,8 | 19,9-17,8 |
| **APC** | p.E941* | 38 | 17 | 667 | 26 | 1038 | 1064 | 2,4 | 3,6-1,3 |
| **APC** | p.A1492Cfs*1513 | 61 | 1373 | 4220 | 2300 | 7940 | 10240 | 22,5 | 23,5-21,4 |
| **BRAF** | p.V600E | 4 | 2757 | 10340 | 4780 | 27480 | 32260 | 14,8 | 15,3-14,3 |
| **FBXW7** | p.A422Qfs*443 | 61 | 2851 | 4349 | 3880 | 6200 | 10080 | 38,5 | 39,6-37,3 |
| **KRAS** | p.G13R | 46 | 1092 | 10223 | 1720 | 25240 | 26960 | 6,36 | 6,74-5,99 |
| **KRAS** | p.G12D | 4 | 8 | 12003 | 10,8 | 27740 | 27750,8 | 0,039 | 0,067-0,011 |
|  |  | 14 | 971 | 3953 | 1296 | 5800 | 7096 | 18,3 | 19,3-17,2 |
|  |  | 38 | 15 | 742 | 20 | 1038 | 1058 | 1,9 | 2,9-1 |
| **KRAS** | p.G12C | 35 | 119 | 6882 | 160 | 11800 | 11960 | 1,35 | 1,59-1,11 |
|  |  | 58 | 418 | 8722 | 6580 | 16660 | 23240 | 28,3 | 29-27,5 |
| **KRAS** | p.G12A | 51 | 3085 | 9370 | 5000 | 20540 | 25540 | 19,6 | 20,2-19 |
| **KRAS** | p.Q61H | 44 | 2274 | 11454 | 3200 | 23960 | 27160 | 11,75 | 12,22-11,28 |
|  |  | 61 | 2852 | 4321 | 4780 | 7680 | 12460 | 38,4 | 39,5-37,2 |
| **KRAS** | p.G12S | 26 | 371 | 2339 | 598 | 4040 | 4638 | 12,9 | 14,4-11,6 |
| **KRAS** | p.G12V | 2 | 1 | 1108 | 1,4 | 1502 | 1503,4 | 0,009 | 0,29-0 |
|  |  | 50 | 7749 | 10601 | 14940 | 24240 | 39180 | 38,1 | 38,8-37,4 |
| **KRAS** | p.G13D | 54 | 13 | 5325 | 18 | 9440 | 9458 | 0,2 | 0,31-0,09 |
| **KRAS** | p.A146V | 1 | 2834 | 6440 | 5240 | 14240 | 19480 | 26,9 | 27,8-26 |
| **NOTCH1** | p.A1104T | 56 | 1008 | 8288 | 1418 | 15420 | 16838 | 8,4 | 8,9-7,9 |
| **NRAS** | p.G12V | 30 | 2353 | 4427 | 4040 | 8280 | 12320 | 32,8 | 33,9-31,7 |
| **NRAS** | p.G12D | 43 | 16 | 4689 | 32 | 11480 | 11512 | 0,27 | 0,4-0,14 |
| **PIK3CA** | p.G1049R | 54 | 1 | 6172 | 1,4 | 10280 | 10281,4 | 0,013 | 0,044-0 |
| **PIK3CA** | p.H1047R | 4 | 5582 | 12790 | 7560 | 23420 | 30980 | 24,4 | 25-23,8 |
| **PIK3CA** | p.E545K | 35 | 232 | 9394 | 288 | 16620 | 16908 | 1,77 | 2-1,54 |
|  |  | 46 | 1496 | 10884 | 2500 | 31320 | 33820 | 7,42 | 8,4-6,9 |
| **PIK3CA** | p.E542K | 50 | 3363 | 10793 | 6100 | 31100 | 37200 | 16,4 | 16,9-15,9 |
| **PIK3CA** | p.F83L | 44 | 3010 | 4561 | 5900 | 9660 | 15560 | 38 | 39,1-36,9 |
| **PIK3R1** | p.S102* | 7 | - | - | - | - | - | - | - |
| **SMAD3** | p.F343L | 28 | 29 | 2708 | 38 | 3780 | 3818 | 0,98 | 1,34-0,62 |
| **TP53** | p.R337C | 56 | 2528 | 7158 | 3920 | 13440 | 17360 | 22,6 | 23,4-21,8 |
| **TP53** | p.M237K | 58 | 4806 | 10442 | 8700 | 26220 | 34920 | 24,9 | 25,6-24,3 |
| **TP53** | p.R110P | 50 | 4054 | 11664 | 6460 | 27600 | 34060 | 19 | 19,5-18,4 |
| **TP53** | p.H214R | 7 | - | - | - | - | - | - | - |
| **TP53** | p.G245S | 51 | 3780 | 8110 | 9000 | 26960 | 35960 | 25 | 25,8-24,3 |
|  |  | 30 | 862 | 2633 | 1560 | 5120 | 6680 | 23,3 | 24,7-21,9 |
| **TP53** | p.R158H | 61 | 3124 | 4627 | 5060 | 7940 | 13000 | 38,9 | 40-37,8 |
| **TP53** | p.R175H | 1 | 4678 | 8065 | 7540 | 15060 | 22600 | 33,4 | 34,2-32,6 |
| **TP53** | p.R248Q | 35 | 192 | 1107 | 216 | 17700 | 17916 | 1,21 | 1,38-1,04 |
|  |  | 43 | 37 | 9454 | 42 | 14060 | 14102 | 0,29 | 0,39-0,2 |
| **TP53** | p.R196* | 26 | 686 | 4023 | 976 | 6400 | 7376 | 13,2 | 14,2-12,3 |
| **TP53** | p.R273H | 28 | 36 | 1984 | 52 | 3060 | 3112 | 1,7 | 2,2-1,1 |

|  |  |  | **Plasma at C3** | | | | | | | | | | | |
| --- | --- | --- | --- | --- | --- | --- | --- | --- | --- | --- | --- | --- | --- | --- |
| Gene | Mutation | Patient | MUT events | WT events | MUT copies/20µL well | | WT copies/20µL well | | TOT copies/ well | | | Fractional Abundance (FA %) | | Poisson 95% C.I |
| **APC** | p.R283* | 43 | 9 | 1461 | 14,2 | | 24200 | | 24214,2 | | | 0,58 | | 0,97-0,19 |
| **APC** | p.Y1376* | 2 | 0 | 710 | 3 | | 1070 | | 1073 | | | 0 | | 0,69-0 |
| **APC** | p.S1032* | 24 | 721 | 2869 | 914 | | 3860 | | 4774 | | | 19,1 | | 20,4-17,8 |
| **APC** | p.E941* | 38 | 18 | 643 | 34 | | 1260 | | 1294 | | | 2,7 | | 3,9-1,4 |
| **KRAS** | p.G12D | 38 | 19 | 764 | 30 | | 1248 | | 1278 | | | 2,4 | | 3,4-1,3 |
| **KRAS** | p.G12C | 58 | 4589 | 6078 | 9340 | | 13380 | | 22720 | | | 41,1 | | 42-40,2 |
| **KRAS** | p.G12V | 2 | 0 | 546 | 0 | | 778 | | 778 | | | - | | - |
| **NRAS** | p.G12D | 43 | 1 | 305 | 3,4 | | 1040 | | 1043,4 | | | 0,3 | | 1,1-0 |
| **PIK3R1** | p.S102* | 7 | 6794 | 6982 | 14460 | | 15020 | | 29480 | | | 49,1 | | 49,9-48,2 |
| **SMAD3** | p.F343L | 28 | 10 | 5169 | 14 | | 8680 | | 8694 | | | 0,16 | | 0,26-0,06 |
| **TP53** | p.M237K | 58 | 5354 | 8181 | 10740 | | 19300 | | 30040 | | | 35,7 | | 36,5-34,9 |
| **TP53** | p.H214R | 7 | 7233 | 7910 | 14580 | | 16540 | | 31120 | | | 46,8 | | 47,6-46 |
| **TP53** | p.R248Q | 43 | 16 | 2414 | 17,6 | | 2820 | | 2837,6 | | | 0,62 | | 0,93-0,31 |
| **TP53** | p.R196* | 28 | 14 | 3769 | 20 | | 6120 | | 6140 | | | 0,33 | | 0,5-0,15 |
|  |  |  | **Plasma at C5** | | | | | | | | | | | |
| Gene | Mutation | Patient | MUT events | WT events | | MUT copies/20µL well | | WT copies/20µL well | | TOT copies/ well | Fractional Abundance (FA %) | | Poisson 95% C.I | |
| **APC** | p.R283* | 43 | 107 | 1966 | | 154 | | 3000 | | 3154 | 4,9 | | 5,8-4 | |
| **APC** | p.R232* | 54 | 6 | 2976 | | 6,8 | | 3620 | | 3626,8 | 0,19 | | 0,34-0,03 | |
| **APC** | p.Y1376* | 2 | 6 | 753 | | 8,2 | | 1062 | | 1070,2 | 0,8 | | 1,4-0,1 | |
| **APC** | p.E941* | 38 | 24 | 1105 | | 36 | | 1700 | | 1736 | 2,1 | | 2,9-1,2 | |
| **KRAS** | p.G12D | 38 | 34 | 945 | | 52 | | 1506 | | 1558 | 3,4 | | 4,5-2,2 | |
| **KRAS** | p.G12V | 2 | 1 | 84 | | 6 | | 444 | | 450 | 1,2 | | 3,9-0 | |
| **KRAS** | p.G13D | 54 | 6 | 2139 | | 8,2 | | 3160 | | 3168,2 | 0,26 | | 0,48-0,05 | |
| **NRAS** | p.G12D | 43 | 99 | 1929 | | 158 | | 3300 | | 3458 | 4,6 | | 5,5-3,7 | |
| **PIK3CA** | p.G1049R | 54 | 0 | 2184 | | 0 | | 3380 | | 3380 | 0 | | - | |
| **SMAD3** | p.F343L | 28 | 4 | 1838 | | 5,2 | | 2480 | | 2485,2 | 0,21 | | 0,42-0 | |
| **TP53** | p.R248Q | 43 | 151 | 2959 | | 166 | | 3500 | | 3666 | 4,5 | | 5,3-3,8 | |
| **TP53** | p.R273H | 28 | 4 | 1618 | | 5,4 | | 2320 | | 2325,4 | 0,23 | | 0,48-0 | |

|  |  |  | **Plasma at C7** | | | | | | |
| --- | --- | --- | --- | --- | --- | --- | --- | --- | --- |
| Gene | Mutation | Patient | MUT events | WT events | MUT copies/20µL well | WT copies/20µL well | TOT copies/ well | Fractional Abundance (FA %) | Poisson 95% C.I |
| **APC** | p.E941* | 38 | 4 | 1525 | 6,4 | 2540 | 2546,4 | 0,25 | 0,5-0 |
| **KRAS** | p.G12D | 38 | 4 | 1448 | 6,2 | 2380 | 2386,2 | 0,26 | 0,53-0 |
| **SMAD3** | p.F343L | 28 | 8 | 1533 | 10 | 1994 | 2004 | 0,5 | 0,85-0,15 |
| **TP53** | p.R273H | 28 | 10 | 1246 | 14 | 1800 | 1814 | 0,77 | 1,25-0,28 |
|  |  |  |  |  |  |  |  |  |  |
|  |  |  |  |  |  |  |  |  |  |
|  |  |  | **Plasma at C9** | | | | | | |
| Gene | Mutation | Patient | MUT events | WT events | MUT copies/20µL well | WT copies/20µL well | TOT copies/ well | Fractional Abundance (FA %) | Poisson 95% C.I |
| **APC** | p.E941* | 38 | 46 | 1950 | 76 | 3440 | 3516 | 2,2 | 2,8-1,5 |
| **KRAS** | p.G12D | 38 | 34 | 1790 | 56 | 3200 | 3256 | 1,7 | 2,3-1,2 |
| **SMAD3** | p.F343L | 28 | 13 | 787 | 16,8 | 1044 | 1060,8 | 1,6 | 2,5-1 |
| **TP53** | p.R273H | 28 | 9 | 564 | 14 | 900 | 914 | 1,5 | 2,6-0,5 |
|  |  |  |  |  |  |  |  |  |  |
|  |  |  | **Plasma at C11** | | | | | | |
| Gene | Mutation | Patient | MUT events | WT events | MUT copies/20µL well | WT copies/20µL well | TOT copies/ well | Fractional Abundance (FA %) | Poisson 95% C.I |
| **APC** | p.E941* | 38 | 15 | 835 | 20 | 1194 | 1214 | 1,7 | 2,6-0,8 |
| **KRAS** | p.G12D | 38 | 5 | 504 | 9,2 | 942 | 951,2 | 1 | 1,8-0,1 |
| **SMAD3** | p.F343L | 28 | 20 | 1073 | 30 | 1666 | 1696 | 1,8 | 2,1-1 |
| **TP53** | p.R273H | 28 | 16 | 885 | 24 | 1398 | 1422 | 1,7 | 2,6-0,9 |
|  |  |  |  |  |  |  |  |  |  |
|  |  |  |  |  |  |  |  |  |  |
|  |  |  | **Plasma at C13** | | | | | | |
| Gene | Mutation | Patient | MUT events | WT events | MUT copies/20µL well | WT copies/20µL well | TOT copies/ well | Fractional Abundance (FA %) | Poisson 95% C.I |
| **APC** | p.E941* | 38 | 17 | 1072 | 26 | 1740 | 1766 | 1,5 | 2,2-0,8 |
| **KRAS** | p.G12D | 38 | 15 | 1002 | 22 | 1580 | 1602 | 1,4 | 2,2-0,7 |
| **SMAD3** | p.F343L | 28 | 11 | 809 | 18 | 1388 | 1406 | 1,3 | 2,1-0,5 |
| **TP53** | p.R273H | 28 | 15 | 657 | 22 | 1026 | 1048 | 2,2 | 3,3-1,1 |

|  |  |  | **Plasma at progression** | | | | | | |
| --- | --- | --- | --- | --- | --- | --- | --- | --- | --- |
| Gene | Mutation | Patient | MUT events | WT events | MUT copies/20µL well | WT copies/20µL well | TOT copies/ well | Fractional Abundance (FA %) | Poisson 95% C.I |
| **APC** | p.Q1367* | 56 | 83 | 1638 | 114 | 2340 | 2454 | 4,6 | 5,6-3,6 |
| **APC** | p.R283* | 43 | 25 | 1412 | 34 | 1980 | 2014 | 1,7 | 2,3-1 |
| **APC** | p.R232* | 54 | 17 | 3799 | 20 | 4980 | 5000 | 0,4 | 0,59-0,21 |
| **APC** | p.Y1376* | 2 | 13 | 946 | 20 | 1532 | 1552 | 1,3 | 2-0,6 |
| **APC** | p.S1032* | 24 | 3298 | 7096 | 5780 | 14860 | 20640 | 28 | 28,8-27,1 |
| **APC** | p.W685* | 30 | 2452 | 5991 | 3720 | 10380 | 14100 | 26,3 | 27,3-25,4 |
| **APC** | p.E941* | 38 | 181 | 1432 | 274 | 2260 | 2534 | 10,8 | 12,3-9,3 |
| **APC** | p.A1492Cfs*1513 | 61 | 1537 | 3995 | 2400 | 6820 | 9220 | 26 | 27,1-24,9 |
| **KRAS** | p.G12D | 38 | 147 | 1320 | 212 | 1960 | 2172 | 9,7 | 11,2-8,2 |
| **KRAS** | p.G12C | 35 | 58 | 2488 | 82 | 3800 | 3882 | 2,1 | 2,7-1,6 |
|  |  | 58 | 6909 | 6138 | 17520 | 14780 | 32300 | 34,2 | 55,1-53,4 |
| **KRAS** | p.Q61H | 61 | 2565 | 3813 | 4380 | 6840 | 11220 | 39 | 40,2-37,8 |
| **KRAS** | p.G12S | 26 | 791 | 2696 | 1288 | 4720 | 6008 | 21,5 | 22,8-20,1 |
| **KRAS** | p.G12V | 2 | 3 | 829 | 5,2 | 1500 | 1505,2 | 0,35 | 0,77-0 |
|  |  | 50 | 6252 | 6846 | 10120 | 11340 | 21460 | 47,1 | 48-46,3 |
| **KRAS** | p.G13D | 54 | 15 | 3063 | 22 | 4840 | 4862 | 0,44 | 0,67-0,22 |
| **NOTCH1** | p.A1104T | 56 | 48 | 1634 | 66 | 2360 | 2426 | 2,7 | 3,5-2 |
| **NRAS** | p.G12V | 30 | 4766 | 6144 | 9540 | 13220 | 22760 | 41,9 | 42,9-41 |
| **NRAS** | p.G12D | 43 | 31 | 1279 | 44 | 1900 | 1944 | 2,3 | 3,1-1,5 |
| **PIK3CA** | p.G1049R | 54 | 0 | 2841 | 0 | 4800 | 4800 | 0 | - |
| **PIK3CA** | p.E545K | 35 | 107 | 3450 | 140 | 5200 | 5340 | 2,7 | 3,2-2,2 |
| **PIK3CA** | p.E542K | 50 | 2039 | 6455 | 3060 | 11500 | 14560 | 21 | 21,9-20,2 |
| **SMAD3** | p.F343L | 28 | 86 | 1934 | 126 | 3000 | 3126 | 4 | 4,9-3,2 |
| **TP53** | p.R337C | 56 | 113 | 1217 | 168 | 1860 | 2028 | 8,2 | 9,7-6,8 |
| **TP53** | p.M237K | 58 | 7535 | 7466 | 18980 | 18720 | 37700 | 50,4 | 51,2-49,5 |
| **TP53** | p.R110P | 50 | 2523 | 6482 | 3640 | 10780 | 14420 | 25,2 | 26,1-24,3 |
| **TP53** | p.G245S | 30 | 2203 | 3673 | 4340 | 7760 | 12100 | 35,9 | 37,1-34,7 |
| **TP53** | p.R158H | 61 | 2600 | 3882 | 4020 | 62080 | 66100 | 39 | 40,2-37,8 |
| **TP53** | p.R248Q | 35 | 108 | 4093 | 126 | 5340 | 5466 | 2,31 | 2,74-1,88 |
|  |  | 43 | 22 | 1849 | 23,6 | 2064 | 2087,6 | 1,13 | 1,6-0,65 |
| **TP53** | p.R196* | 26 | 1660 | 5073 | 2032 | 6860 | 8892 | 22,9 | 23,8-21,9 |
| **TP53** | p.R273H | 28 | 106 | 1347 | 156 | 2060 | 2216 | 7 | 8,3-5,7 |

**Table S8.** The tables list the ddPCR events (mutant and wild type cases) obtained by analyzing serial plasma samples at different time points (baseline until progression) of 20 aCRC patients. Total number of copies per sample, Fractional Abundance (FA %) and Poisson CI. (95%) are shown.
